# Two Kinds of Turnover: Contrasting Drivers of Beta Diversity Between Cultivated and Spontaneous Plants Along a Coastal Island-to-Mainland Gradient

**DOI:** 10.3390/biology15211946

**Published:** 2026-10-28

**Authors:** Hongjun Zhou, Luying Ye, Yahong Xing, Runsheng Zhang, Haixia Lu, Linting Zhang, Rongli Liao, Ruiyi Jiang, Libin Zhuang, Chuanyuan Deng

**Affiliations:** 1 College of Landscape Architecture and Art, Fujian Agriculture and Forestry University, Fuzhou 350002, China; 2 Fuzhou Urban Ecosystem Research Station in Fujian, Fuzhou 350012, China; 3 Island Research Center, Ministry of Natural Resources, Pingtan 350400, China

**Keywords:** beta-diversity, turnover, nestedness, urban parks, coastal gradient, cultivated and spontaneous vegetation

## Abstract

Urban park plant communities comprise cultivated and spontaneous species, but whether their beta-diversity drivers differ and whether urbanization homogenizes or differentiates composition remains unclear. We surveyed 102 plots in 21 parks along a coastal island-to-mainland gradient in Fuzhou, China. We partitioned taxonomic, functional and phylogenetic beta-diversity into turnover and nestedness, related them to environmental, anthropogenic and spatial predictors using Mantel tests, variation partitioning and db-RDA, and modeled local contributions (LCBD) using mixed models and structural equations. Turnover dominated both groups, with higher turnover and lower nestedness in cultivated plants. Cultivated functional and phylogenetic nestedness rose with distance (*p* < 0.01), whereas spontaneous nestedness fell across all dimensions (*p* < 0.05); the slopes differed significantly in all three dimensions. Taxonomic beta-diversity exceeded functional and phylogenetic, and LCBD synchronization was taxonomic only. Landscape configuration and anthropogenic factors drove cultivated beta-diversity, whereas spontaneous beta-diversity related to spatial structure, park area and elevation. Marine exposure lost significance for most responses once space was controlled. Composition varied among regions; island plots the most concentrated; LCBD depended on park area for spontaneous plants and on landscape configuration for cultivated ones. The two kinds of turnover reflect different processes, ecological replacement and planting decisions, and require parallel management.

## Simple Summary

Urban parks contain two kinds of plants: those that people plant, and those that arrive on their own. We asked whether these two groups change in composition from park to park for the same reasons. We recorded all plants in 102 small survey plots across 21 parks, from an island on the coast to the inland city of Fuzhou, China, and compared the planted and the self-established species. Both groups changed mainly because species were replaced by different species, rather than because some plots simply held fewer species. For the planted group, this replacement was stronger, and the differences among parks were explained mostly by the surrounding landscape and by human activity such as planting and management. For the self-established group, differences were explained mostly by location, park size and elevation. Distance from the sea mattered little for most patterns once location was accounted for. The two groups therefore follow different rules: one reflects decisions about what to plant, the other reflects natural colonisation. Park managers should treat them separately, choosing and arranging planted species with regional variety in mind while protecting the places where wild plants establish, so that cities keep a richer and more varied plant community.

## 1. Introduction

Urban ecosystems harbor substantial biodiversity, provide critical ecosystem services to nearly half of the global population, and have become a focus of contemporary ecological and conservation research [1,2,3]. Evidence at the global scale indicates that urbanization is systematically reshaping plant diversity. A review of 922 studies shows that urban growth already exerts substantial direct and indirect impacts on habitats and biodiversity, and that the indirect impacts act over broader extents yet remain markedly understudied [4]. Across a dataset covering 110 cities in 36 countries, the spontaneous flora of cities retains only about one-quarter of the native flora, and anthropogenic factors explain diversity loss better than natural factors; nevertheless, cities still harbor locally endemic species, offering opportunities for biodiversity conservation at regional and global scales [5]. Urban habitat type explains floristic composition better than climate, with native and alien species responding differently [6].

Assessments and conservation targets for urban biodiversity currently rely on species richness as a proxy [7,8,9]. Species richness, however, cannot serve as a proxy for the other dimensions of diversity. High species richness in urban plants does not necessarily come with corresponding phylogenetic diversity [10], and the spatial patterns of taxonomic, phylogenetic, and functional diversity are often mismatched, so conservation planning must integrate them rather than treat one as a substitute for another [11]. Regional diversity comprises two components: local (alpha) diversity and differentiation among communities (beta) [12], with beta diversity representing the component that accumulates from compositional differences among local communities [13,14]. Beta diversity cannot be inferred from alpha diversity [15]. Beta diversity therefore links local monitoring to regional conservation and carries information value for conservation planning that is independent of species richness [9].

Beta diversity can be decomposed into turnover and nestedness components. Turnover corresponds to the replacement of species identities among plots, whereas the nestedness component corresponds to richness differences (i.e., species loss); the two arise from different processes and point to different mechanisms and management actions [16,17]. A meta-analysis across organism groups shows that turnover is usually the dominant component of total beta diversity, although its relative importance varies with scale and taxon [18]. The decomposition approach itself also affects the detection of homogenization signals [19].

Plant communities in urban parks are composed of both cultivated and spontaneous species, and the occurrence of each is determined by different processes. The occurrence of cultivated plants depends on introduction and planting decisions, and their composition approximates the commercial horticultural supply pool in taxonomic and phylogenetic terms; spontaneous plants depend on environmental filtering and natural regeneration, and their composition is closer to that of vegetation in natural areas [20,21]. The composition and functional types of cultivated vegetation are reshaped by human selection and differ markedly from natural remnant vegetation in both species and functional types [22], while plant diversity in parks is driven jointly by natural and anthropogenic factors [23]. The beta diversity of the two groups therefore records compositional differences formed by different processes, and their turnover and nestedness patterns and drivers need to be compared in parallel.

One ecological consequence of urbanization is biotic homogenization [24,25]. Homogenization refers to the increase in similarity among floras over time [26]; a single survey can test only spatial homogenization at one point in time [27], and the evidence at this level points in inconsistent directions. Evidence is mixed: invasive and archaeophyte groups are compositionally similar among distant cities [28], German urban floras show homogenization only for native and pre-1500 alien species, with post-1500 aliens differentiated [29], and the similarity of urban forests across 38 cities does not change with the inclusion of alien species [30]. At the plot level, by contrast, the beta diversity of spontaneous plants increases with urbanization, indicating compositional differentiation [31]. A systematic assessment of 225 studies shows that empirical support for urban biotic homogenization stands at only 55%, and that the evidence is heavily biased toward the taxonomic dimension, with homogenization at the genetic and functional levels having long been neglected [26,32]. In addition, most existing studies of urban beta diversity focus on spontaneous plants [33], whereas homogenization of the cultivated group has been confirmed across cities [34]. Continental-scale comparisons of residential systems show that lawns are more similar to one another than reference vegetation in both cultivated and spontaneous plant composition, while species richness varies more among lawns [35,36], and yard-scale studies have likewise characterized the composition and homogenization of the cultivated and spontaneous components separately [20,37]. However, no study has yet decomposed the three dimensions of beta diversity in parallel for the two groups, or contrasted their turnover and nestedness components, at the scale of park plots within a single environmental gradient.

Translating compositional differences into management action requires apportioning beta diversity to specific locations. The local contribution to beta diversity (LCBD) measures the contribution of a single plot to regional beta diversity [38] and can identify the locations that contribute most to regional compositional differences, thereby providing operational targets for conservation and management priorities [9].

Coastal island-to-mainland gradients provide a strong filtering setting for testing the questions outlined above. Salt spray, wind, and human activity vary in concert along the coast-to-inland direction, forming a strong gradient of stress and disturbance [39]; plant diversity patterns along the subtropical coast of China vary with distance from shore [40], and cultivated species diversity in urban parks on coastal islands also increases with distance from shore [41]. In such settings, however, marine exposure and spatial location are highly collinear, and any attribution of beta diversity must explicitly account for spatial structure [42,43]. In Fuzhou, the Island region (Pingtan), the Coastal region (Changle), and the Inland region (the main urban district) are arrayed in sequence along the coast-to-inland direction, forming a complete coastal gradient in which marine exposure and spatial structure can be estimated separately. This gradient provides an ideal setting for comparing the beta diversity of the two groups in parallel.

In summary, three questions remain: whether the beta diversity of cultivated and spontaneous plants is driven by the same processes, whether urbanization makes community composition converge or diverge, and whether the local contributions of the two groups point to the same locations. To address them, we compared the turnover and nestedness patterns and the driver structure of beta diversity between cultivated and spontaneous plants across 102 plots in 21 parks along the coastal island-to-mainland gradient in Fuzhou, and apportioned beta diversity to the plot level to identify the sources of local contributions and their management implications.

## 2. Materials and Methods

### 2.1. Study Area and Sampling Design

**Figure 1:**
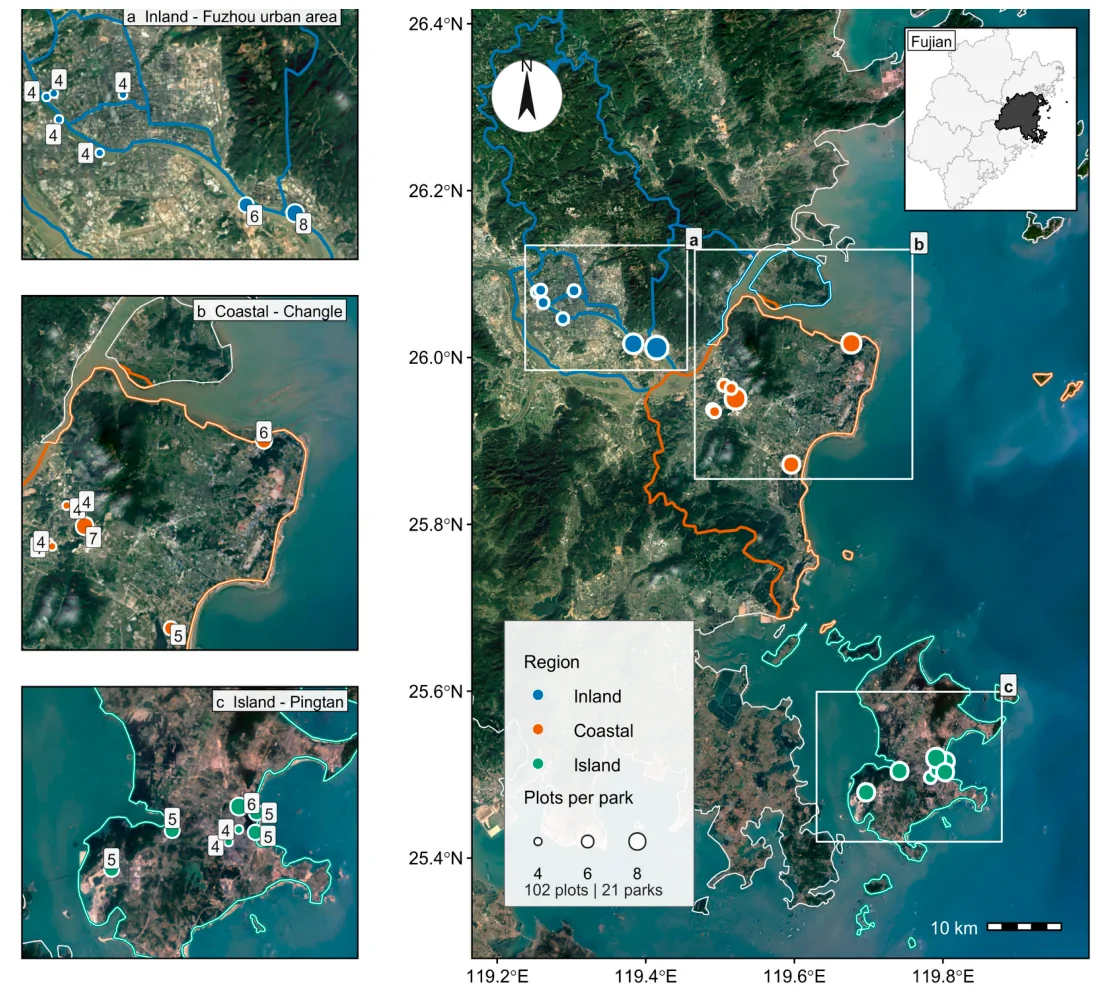
Study area and plot distribution. The 21 urban parks lie along the coastal island-to-mainland gradient in Fuzhou, China. Points are colored by region: the Inland region (Inland, blue), the Coastal region (Coastal, orange) and the Island region (Island, green); point diameter indicates the number of plots per park (4–8). Each region contains seven parks and 34 plots, for a total of 102 plots. Panels (**a**–**c**) are enlargements of the three regions ((**a**), Inland region, the main urban district of Fuzhou; (**b**), Coastal region, Changle; (**c**), Island region, Pingtan). The inset in the upper right corner shows the location of the study area within Fujian Province. Coordinates are in WGS 84.

The study area is located along the coast of Fuzhou, Fujian Province, China. From the interior toward the sea, it comprises the Inland region (the main urban district of Fuzhou), the Coastal region (Changle District) and the Island region (Pingtan Island), forming a natural island–mainland environmental gradient along which marine stresses such as salt spray and wind decrease from the coast inland (Figure 1). This study was based on a single survey, in which 21 urban parks were selected by stratified random sampling; four to eight plots were established in each park, for a total of 102 plots. Each plot was surveyed in three layers (tree, shrub and herb), represented by one 20 m × 20 m plot, four 5 m × 5 m plots and five 1 m × 1 m plots, respectively; species identity, abundance and growth indicators were recorded. For the diversity analyses, records were converted to presence/absence (incidence) at the plot level, and no cover or abundance-based metrics were used, because abundance is not comparable between the two plant groups (Section 2.2). The surveys were completed between 26 May and 6 July 2026, following the regional sequence Island, Inland and then Coastal, with one park surveyed per day (Table S10). The plant material used in this study was identified and recorded in the field, and no rare or endangered species and no material requiring specific collection permits were involved; all field surveys were conducted in urban parks with the permission of the relevant park authorities and complied with the relevant national and local regulations and with the guidelines of Fujian Agriculture and Forestry University.

All 21 parks are public green spaces whose routine maintenance is organized by the local public green-space authorities. Standard operations comprise pruning, weeding, fertilization and replanting, watering, pest and disease control, and litter and green-waste removal. Maintenance is delivered either directly by the managing departments or by service companies contracted through public bidding, with watering applied as required—mainly by mobile water trucks—and sustained irrigation concentrated on new plantings and seasonal flower beds. Maintenance nevertheless differed among parks: parks in peri-urban or township settings, such as Jiangjunshan Park in Changle—where maintenance is concentrated in the core area, with adjacent zones still under renovation and directly bordering residential properties—received less intensive pruning and clearing than most parks, although its composite intensity index is not low, because trampling and accessibility scored highest among all parks (Section 2.4).

### 2.2. Classification of Species Records and Incidence Matrices

Each species record was classified as cultivated or spontaneous according to whether it involved human selection or disturbance, so that the species list of each plot was divided into cultivated and spontaneous subsets. Cultivated individuals were planted, whereas spontaneous individuals occurred naturally; the same species could carry both types of record. Seedlings of cultivated trees appearing in the shrub layer were recorded as spontaneous, because they represent natural regeneration rather than cultivated supply [44]. Species were identified following Flora of China, and all names were checked against the World Flora Online database to ensure a consistent nomenclature. Plot-by-species abundance matrices were constructed separately for the spontaneous and the cultivated records and were then binarized to obtain the incidence matrices (306 spontaneous species and 250 cultivated species, of which 35 had both types of record). Individuals were counted in all three layers; however, individual numbers in the herb layer were orders of magnitude higher than in the tree and shrub layers, and the cultivated group was dominated by trees whereas the spontaneous group was dominated by herbs. Abundance is therefore not comparable between the two groups, and abundance-weighted metrics would be dominated by the counts of herb individuals; all beta diversity, LCBD and species contribution to beta diversity (SCBD) calculations were therefore based uniformly on the incidence matrices.

### 2.3. Beta Diversity and Component Decomposition

Beta diversity was computed for the taxonomic, functional and phylogenetic dimensions of both plant groups, in all cases from incidence data. The three dimensions followed the same decomposition, partitioned into turnover and nestedness components according to the Baselga framework [45].

(1) $$\beta_{\mathrm{sor}}=\frac{b+c}{2a+b+c}$$

(2) $$\beta_{\mathrm{sim}}=\frac{\min(b,\;c)}{a+\min(b,\;c)}$$

*β*_sne_ = *β*_sor_ − *β*_sim_ (3)

(4) $$A_{\mathrm{ij}}=\sum_{k}l_{k}\cdot I_{k}(i)\cdot I_{k}(j)$$

In Equations (1)–(4), a is the number of diversity units shared by two plots, and b and c are the numbers of diversity units occurring in only one of the two plots; the unit is the species in the taxonomic dimension and the branch length in the functional and phylogenetic dimensions. Equation (4) gives the branch-length computation of the shared component, where lk is the length of branch k in the functional or phylogenetic tree and Ik(i) is an indicator function that equals 1 when the species list of plot i covers at least one terminal branch below branch k and 0 otherwise; PDi = Σk lk Ik(i) is the total branch length covered by plot i, and b = PDi − Aij and c = PDj − Aij. βsim measures the replacement of diversity units, βsne measures the nestedness component, and the turnover proportion is computed as βsim/βsor. In the taxonomic dimension, units were species and the implementation of the betapart package was used [46]; in the functional and phylogenetic dimensions, units were branch lengths computed following Equation (4), and the species subsets of each group were expanded to the full width of the corresponding tree (absent species coded as 0) so that beta diversity was on the same scale in the two groups. The functional dimension used three leaf-economics-spectrum traits: specific leaf area (SLA), leaf area (LA) and leaf dry matter content (LDMC). These three leaf traits represent the leaf economic spectrum, so the functional results primarily describe differentiation in leaf economic strategies rather than the complete functional niche of the species. Trait values were compiled and imputed by combining field measurements, measurements made by our research group, the BIEN database, genus-level means and k-nearest-neighbor (kNN) niche prediction; low-frequency species occurring in too few plots (63 species) and species still missing at least one trait after imputation (57 species) were excluded. The remaining 401 species had complete values for all three traits, retaining 216 of the 306 spontaneous species and 219 of the 250 cultivated species. The phylogenetic dimension retained the species with records in GBOTB: 305 of the 306 spontaneous species, the single excluded species being a non-target group (the moss *Funaria hygrometrica*); for the cultivated group, the 250 species entries became 238 tree tips after cultivars were merged into their parent species, and no species was excluded for lack of a GBOTB record. The functional tree was built from the complete trait table of the 401 species by UPGMA clustering of Gower distances, and the phylogenetic tree was built with V.PhyloMaker2 from the GBOTB backbone (507 terminal taxa in total after merging the two groups; [47]). Beta diversity and its turnover and nestedness components all entered the subsequent analyses.

### 2.4. Spatial Variables and Environmental Factors

The spatial distance matrix was the geodesic distance (km) among the WGS 84 coordinates of the plots. Spatial variables were distance-based Moran’s eigenvector maps (dbMEM; [42,48]), truncated at the longest edge of the minimum spanning tree; only eigenvectors with positive spatial autocorrelation were retained and entered the models after forward selection [49].

The candidate pool of environmental factors contained 23 variables in nine categories (full definitions, units, hierarchy and data sources in Table S1): management intensity; slope, elevation and eight-directional aspect; soil pH, soil water content, electrical conductivity and corrected soil temperature; canopy closure and the normalized difference vegetation index (NDVI) at 1 km; precipitation of the driest month (WorldClim 2.1; [50]); the marine stress factor and the sea-facing index; the landscape principal components Landscape PC1 (1 km), Landscape PC1 (3 km) and Landscape PC2 (3 km); nighttime lights, population density, distance to road and motor access; and park area, shape index and park age. The marine stress factor operationalizes marine exposure: it was synthesized as the first axis of a principal component analysis (PCA) of 16 marine variables (salt-spray aerosol concentrations, wind fields and sea-breeze frequency from the MERRA-2 reanalysis), which explained 93.4% of the variance; higher values indicate stronger marine exposure. For the cultivated group, canopy closure and NDVI were removed, because the cultivated canopy is determined by planting and thus shares the same origin as cultivated supply, leaving 21 variables. Collinearity among the variables entering each model was verified with variance inflation factors (VIF; maximum 1.92, all below 2).

Management intensity (MF) was scored in each plot on three disturbance dimensions—trampling and accessibility, pruning and clearing intensity, and proximity to built facilities—each rated from 1 (weak) to 4 (strong disturbance). The three standardized scores were reduced to their first principal component (55.9% of the variance explained; all loadings positive), yielding a plot-level index in which higher values indicate stronger management intensity. MF ranged from −2.50 to 2.91 across the 102 plots (mean 0.00, SD 1.30); park-level means ranged from −1.27 to 1.46, and the mean within-park range was 2.52 (0.57–4.09), indicating that management intensity varied both among and within the 21 parks.

Landscape PC1 is the first axis of two separate principal component analyses of landscape-configuration metrics: at the 1-km scale (plot-level predictors), 26 metrics explain 48.5% of the variance, and at the 3-km scale (park-level predictors), 27 metrics explain 43.6%. At both scales, higher values indicate a more aggregated, less fragmented landscape, and lower values a more fragmented landscape with higher edge and patch density.

### 2.5. Plot-Level Local Contributions and Species Contributions

The LCBD and the SCBD were computed following the definitions of Legendre and De Cáceres [38]. The LCBD was derived from the dissimilarity matrix of each dimension (Sørensen dissimilarity for the taxonomic dimension; branch-length dissimilarity for the functional and phylogenetic dimensions), and the taxonomic results agreed with the output of adespatial::beta.div; the SCBD was computed from incidence data in the taxonomic dimension by decomposing the total sum of squares among species.

(5) $${\mathrm{LCBD}}_{i}=\frac{\sum_{j\neq i}D_{\mathrm{ij}}^{2}}{2\sum_{i<k}D_{\mathrm{ik}}^{2}}$$

In Equation (5), Dij is the Sørensen dissimilarity between plots i and j. LCBD values range from 0 to 1 and sum to 1 across all plots, measuring the contribution of an individual plot to regional beta diversity; the SCBD decomposes the total sum of squares among species, measuring the contribution of an individual species to regional beta diversity. The LCBD was log-transformed for the univariate and factor-screening models and logit-transformed for the piecewise structural equation models; because LCBD values lie far from the 0 and 1 boundaries, the two transformations are nearly equivalent over this range.

### 2.6. Statistical Inference

Plots were nested within parks and parks within regions; permutations for the permutational multivariate analysis of variance (PERMANOVA), for tests of the homogeneity of multivariate dispersions (betadisper) and for distance-based redundancy analysis (db-RDA) were therefore always performed at the level of whole parks (21 parks; 999 permutations), so that significance would not be inflated by freely permuting plots [51]. The permutation scheme reassigned region labels among the 21 parks while keeping the within-park plot structure intact. Mantel and partial Mantel tests used 9999 permutations, giving a minimum attainable *p* of 0.0001, whereas the remaining permutation tests used 999 permutations, giving a minimum attainable *p* of 0.001; the species-based permutation tests for LCBD used 9999 permutations, as described below. Functional and phylogenetic dissimilarities are non-Euclidean semimetrics; before db-RDA and variation partitioning, each distance matrix was converted into Euclidean coordinates by principal coordinates analysis (PCoA), retaining only the axes with positive eigenvalues [52]. The Lingoes correction and uncorrected db-RDA were not adopted because the low-variance components were numerically unstable.

The Mantel and partial Mantel tests followed a design of three mutually exclusive blocks: the spatial block S (geodesic distances), the non-marine environmental block E (21 factors for the spontaneous group and 19 for the cultivated group) and the marine exposure block M (the marine stress factor, the sea-facing index and log_10_-transformed distance to the coastline). Block E consisted of the candidate factors remaining after the removal of the marine stress factor and the sea-facing index; log_10_-transformed distance to the coastline belonged exclusively to block M, so the three blocks did not overlap. The three blocks were tested against the 18 beta diversity responses (two plant groups × three dimensions × three beta components) with simple Mantel tests and pairwise partial Mantel tests, giving 162 tests in total (9999 permutations); all *p* values were corrected for the false discovery rate (FDR) with the Benjamini–Hochberg (BH) method [53]. Distance decay was quantified by regressing beta diversity (pairwise dissimilarities) on geographic distance, reporting the slope b per 100 km; b > 0 indicates that the component increases with distance. Inference was based on whole-park permutation tests.

Variation partitioning used the environmental variables and the spatial variables (the dbMEM variables retained by forward selection) as explanatory blocks and reported the adjusted R^2^ (R^2^_adj_); the significance of the pure environmental and pure spatial fractions was tested with 999 whole-park permutations. The db-RDA reported the adjusted R^2^ of the full model together with its permutation test. The full model included all environmental factors of the candidate pool (23 for the spontaneous group and 21 for the cultivated group) plus the two dbMEM variables retained by forward selection, giving 25 and 23 predictors in total. Forward selection followed the two stopping criteria of Blanchet et al. (2008) and yielded the minimal sufficient set of variables [49]. Marginal effects were computed within the full model as the ΔR^2^ of a single factor after accounting for all remaining factors (Type III sums of squares); permutations followed the hierarchy of the factors, with park-level factors permuted as whole parks and plot-level factors permuted freely, and the resulting *p* values were FDR-corrected. Technically, the park-level permutations were implemented with an explicit permutation matrix: in each permutation, the 21 park blocks were shuffled while the plots within each park kept their original order, so that park-level factors were reassigned among whole parks, whereas plot-level factors were permuted freely across the 102 plots. Forward selection and marginal effects address different questions: the former identifies the smallest set of variables needed to reach a comparable explanatory power, whereas the latter tests whether an individual factor carries information that cannot be replaced by the other variables. When predictors are correlated, a factor may therefore be significant in the marginal-effects analysis without being retained by forward selection, and vice versa.

Regional differences and continuous gradients in LCBD were tested with linear mixed models (LMMs), using the log-transformed LCBD as the response, park as a random intercept and Kenward–Roger degrees of freedom [54]; pairwise comparisons among regions were Holm-corrected. Factor screening began with bidirectional stepwise selection based on the corrected Akaike information criterion (AICc), with the marine stress factor forced into the model; reported results were based primarily on model averaging across AICc-ranked subsets, with a maximum of eight variables per subset, and on hierarchical partitioning (glmm.hp). The significance of LCBD was assessed with 9999 species-based permutations, with the 102 plots Holm-corrected, and was compared against a within-park stratified null model that preserved the species composition of each park. One piecewise structural equation model (piecewise SEM) was built for each dimension (taxonomic, functional and phylogenetic; piecewiseSEM package; [55]); the LCBD of the cultivated group entered the equations of the spontaneous group as an endogenous variable in order to estimate cross-group pathways; each equation included a park random intercept, and overall model fit was assessed with Fisher’s C tests.

Permutation-based tests do not assume normality. Residual normality of the LMMs was checked graphically; collinearity in the multivariate models was verified with VIF (all below 2); and spatial dependence was modeled explicitly with the dbMEM variables. All analyses were performed in R 4.4.3, mainly using the packages betapart, adespatial, vegan, lme4, lmerTest, piecewiseSEM, glmm.hp, MuMIn, ape, cluster, V.PhyloMaker2, spdep, permute and missForest (see Supplementary Materials, Table S9 for version numbers).

During the preparation of this work, the authors used DeepSeek Harness (DeepSeek-V4) to assist with internal consistency checking (cross-verification of numerical values reported in figures and tables against the main text, and of in-text citations against the reference list) and language editing. The tool was not used for study design, data analysis, interpretation of results, or the formation of conclusions. The authors have reviewed and verified all outputs against the original data and take full responsibility for the content of this publication.

## 3. Results

### 3.1. Decomposition of Beta Diversity into Turnover and Nestedness Components

Across the 21 parks, 306 spontaneous and 250 cultivated taxa were recorded, of which 35 had both cultivated and spontaneous records. Plot-level richness ranged from 1 to 48 taxa for spontaneous plants and from 2 to 29 for cultivated plants, and park-level richness ranged from 18 to 104 and from 9 to 70, respectively (Table S2). The numbers of taxa recorded were largest in the Inland region for both groups (189 spontaneous and 189 cultivated taxa) and smallest in the Island region (116 and 65), with the Coastal region intermediate (162 and 126). The spontaneous taxa occurring in the largest numbers of parks were *Cyperus rotundus* (19 of 21 parks), *Oxalis pes-caprae*, *Youngia japonica*, *Bidens alba* and *Digitaria sanguinalis* (18 each) and *Erigeron canadensis* (16 parks); among cultivated taxa, the lawn grasses *Cynodon dactylon* (20 parks) and *Zoysia japonica* (18) and the woody species *Ficus microcarpa* (18) and *Duranta erecta* (17) were the most widespread.

**Figure 2:**
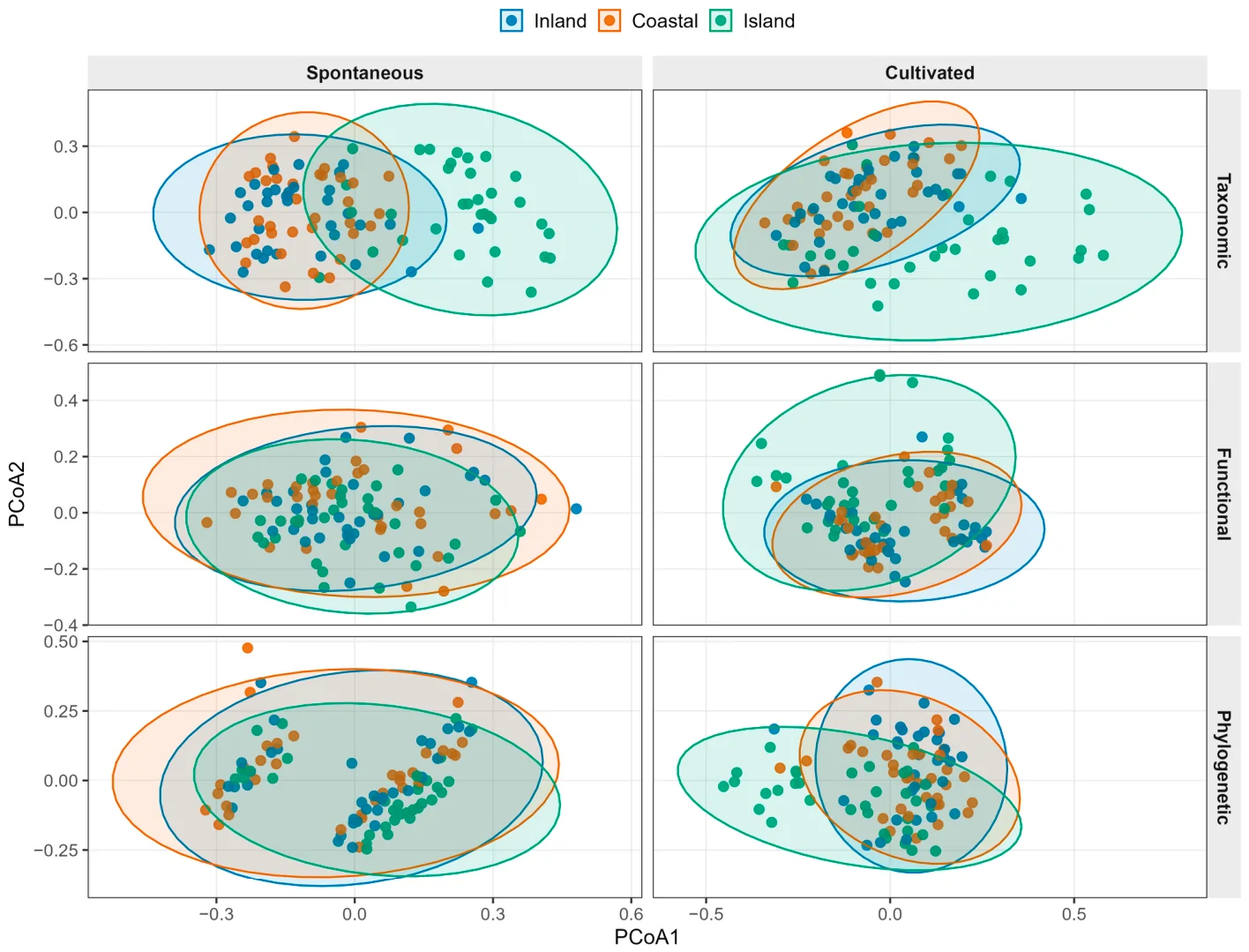
Ordination of the community composition of the plots in the three regions. Ordinations are based on principal coordinates analysis (PCoA) of the dissimilarity matrix of each dimension (Sørensen dissimilarity for the taxonomic dimension; branch-length dissimilarity for the functional and phylogenetic dimensions). The left column shows spontaneous plants and the right column cultivated plants. Points are individual plots, colored by the Inland, Coastal and Island regions; ellipses are 95% confidence ellipses of each region (34 plots per region).

The decomposition of beta diversity (Table 1) showed that beta diversity in both groups was dominated by the turnover component: turnover proportions were 0.655–0.889 in the spontaneous group and 0.776–0.929 in the cultivated group, nestedness values were 0.059–0.169, and the turnover proportion was higher in the cultivated group than in the spontaneous group in all three dimensions. Total taxonomic beta diversity was the highest (0.837 in the spontaneous group and 0.825 in the cultivated group), whereas total functional and phylogenetic beta diversity ranged from 0.488 to 0.541. Paired tests showed that total beta diversity differed significantly between the two systems in the taxonomic (+0.012) and phylogenetic (−0.021) dimensions but not in the functional dimension (*p* > 0.05); the differences in the turnover component were negative and those in the nestedness component positive in all three dimensions (magnitudes of 0.02–0.08), and all of these differences were significant (*p* = 0.001) with the exception of total beta diversity in the functional dimension, indicating that nestedness was significantly lower in the cultivated group. The magnitude of the systematic difference in total beta diversity was extremely small (no more than 0.021), representing a difference that is statistically detectable but negligible; moreover, the sign of the difference in the taxonomic dimension reverses on the common species set. We therefore report this difference without ecological interpretation. A sensitivity analysis based on the common species set showed that the systematic differences in turnover proportion and in the nestedness component, as well as the pattern of taxonomic beta diversity exceeding functional and phylogenetic beta diversity, were robust; only the systematic difference in total beta diversity of the taxonomic dimension was sensitive to species coverage (sign reversal on the trait set, magnitude not exceeding 0.02). PERMANOVA tests among the three regions (Table 2) showed that all six responses (three dimensions in each of the two systems) were significant (*p* < 0.01); differences in dispersion were limited, with only the spontaneous taxonomic and the cultivated phylogenetic responses reaching significance (*p* < 0.05). Dispersion was highest in the Inland region for all six responses; among these, it was lowest in the Island region for the three spontaneous dimensions and for the cultivated phylogenetic dimension (Table 2). Ordinations of community composition among the three regions (Figure 2) showed that plots of the three regions overlapped overall; clear separation of the Island-region plots appeared only in all three dimensions of beta diversity of the cultivated group and in the spontaneous taxonomic dimension. This deviation was not pronounced between the Inland and Coastal regions, and no continuous gradient was apparent along the island-to-coast-to-inland direction.

### 3.2. Mantel and Partial Mantel Tests of Beta Diversity

**Figure 3:**
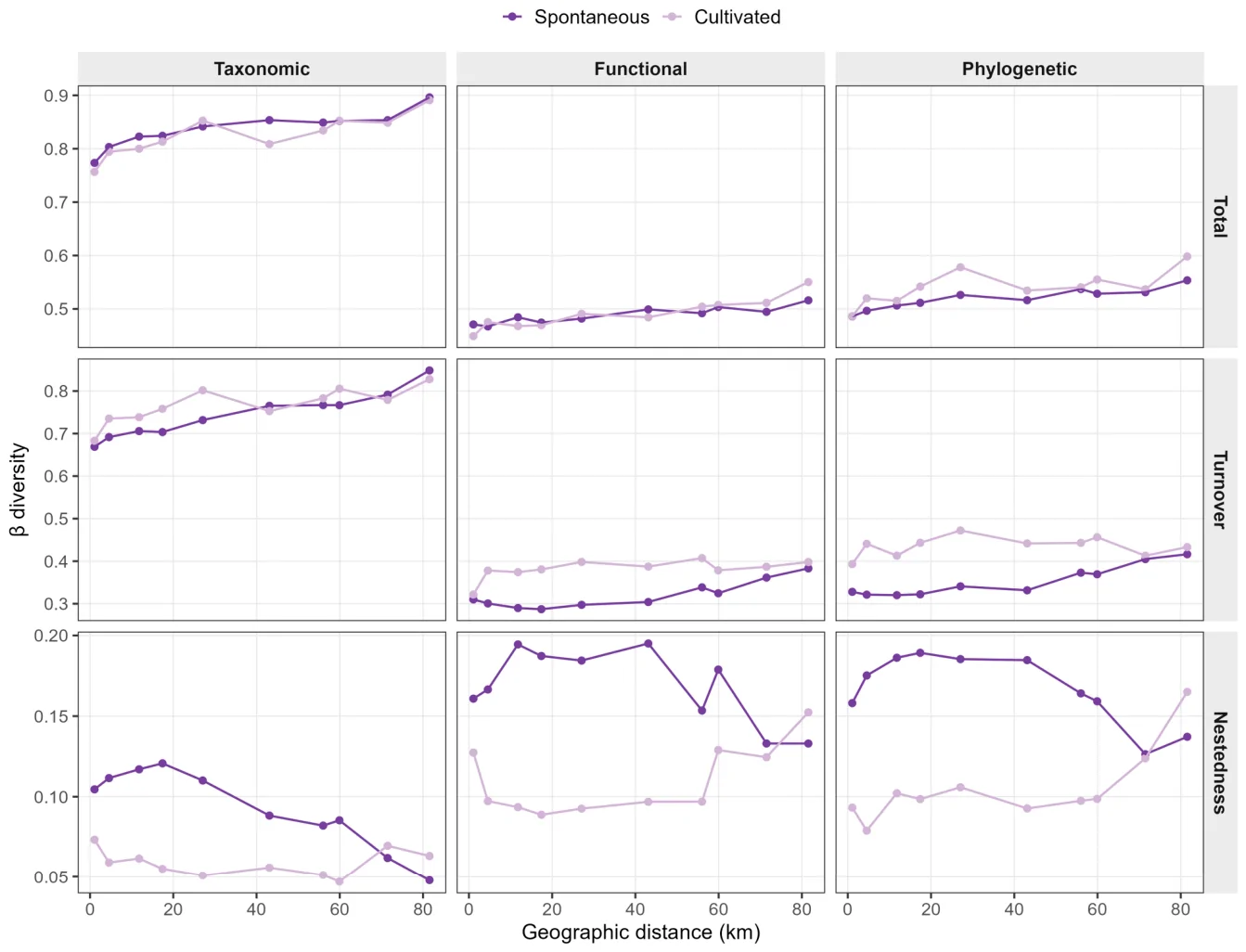
Variation in total beta diversity and its turnover and nestedness components with geographic distance, shown separately for the taxonomic, functional and phylogenetic dimensions of the spontaneous and cultivated groups.

**Figure 4:**
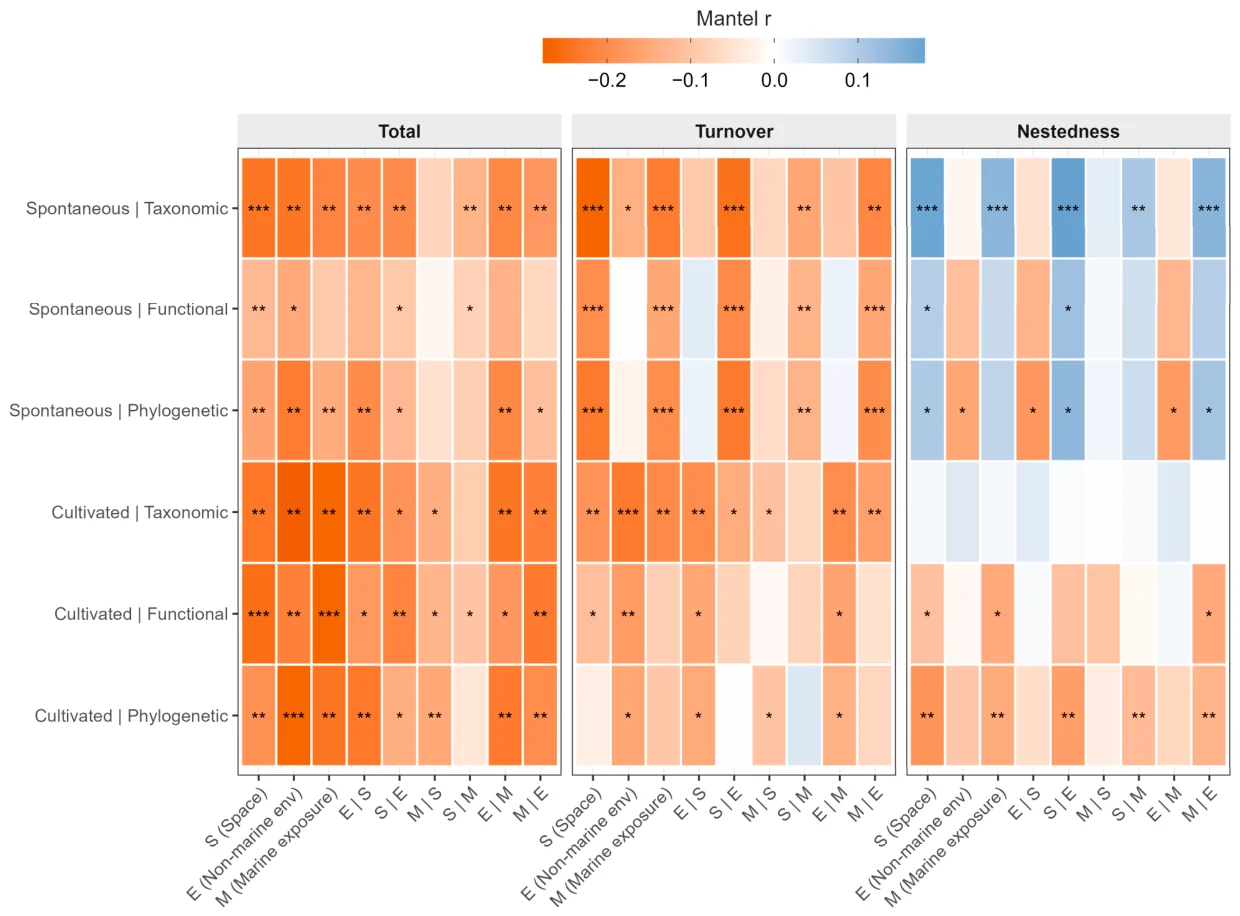
Correlation coefficients from Mantel and partial Mantel tests relating beta diversity to the three distance matrices (spatial distance, the non-marine environment and marine exposure). Panels are arranged by component (Total, Turnover, Nestedness). Row labels give the plant group and the diversity dimension; column labels give the distance matrices: S, spatial distance; E, non-marine environment; M, marine exposure; a label of the form X|Y denotes the partial Mantel test of matrix X while controlling for matrix Y. ***, ** and * denote q < 0.001, q < 0.01 and q < 0.05, respectively, after false discovery rate (FDR) correction.

Distance-decay analyses (Figure 3) showed that total beta diversity increased with geographic distance in all three dimensions, and that the turnover component did so in five of the six responses, with the largest increase for taxonomic beta diversity. The direction of the nestedness component was opposite in the two groups: it decreased with distance in all three dimensions of the spontaneous group (b = −0.048 to −0.078, *p* < 0.05) and increased with distance in the functional and phylogenetic dimensions of the cultivated group (b = 0.043 to 0.058, *p* < 0.01); no distance trend was detected in the cultivated taxonomic dimension (*p* > 0.05). The difference in nestedness slopes between the two groups was significant in all three dimensions (taxonomic dimension, *p* < 0.01; functional and phylogenetic dimensions, *p* < 0.001; Table S8). Mantel tests (Figure 4) showed that, except for spontaneous functional beta diversity, total beta diversity was significantly correlated with all three types of distance (spatial distance, marine exposure and the non-marine environment; q < 0.05), and the correlations were generally stronger in the cultivated group than in the spontaneous group; the significant patterns of the turnover component resembled those of total beta diversity, whereas significant correlations of the nestedness component occurred for several responses. Partial Mantel tests showed that, after controlling for spatial distance, the correlations with marine exposure were no longer significant for most responses and were retained only for a few responses of the cultivated group (q < 0.05), whereas after controlling for the non-marine environmental distance, the correlations with spatial distance remained significant for most responses (q < 0.05). Variation partitioning gave directionally consistent results: pure environmental effects were significant for total beta diversity and for the turnover component (R^2^_adj_ = 0.062–0.173, *p* < 0.05), whereas the pure spatial effect was significant only for the turnover component of cultivated taxonomic beta diversity (R^2^_adj_ = 0.015). For total beta diversity, pure environmental effects were higher in the cultivated group (R^2^_adj_ = 0.148–0.170) than in the spontaneous group (R^2^_adj_ = 0.100–0.104); neither effect was significant for the nestedness component (*p* > 0.05; Figure S1).

### 3.3. Drivers of Beta Diversity

**Figure 5:**
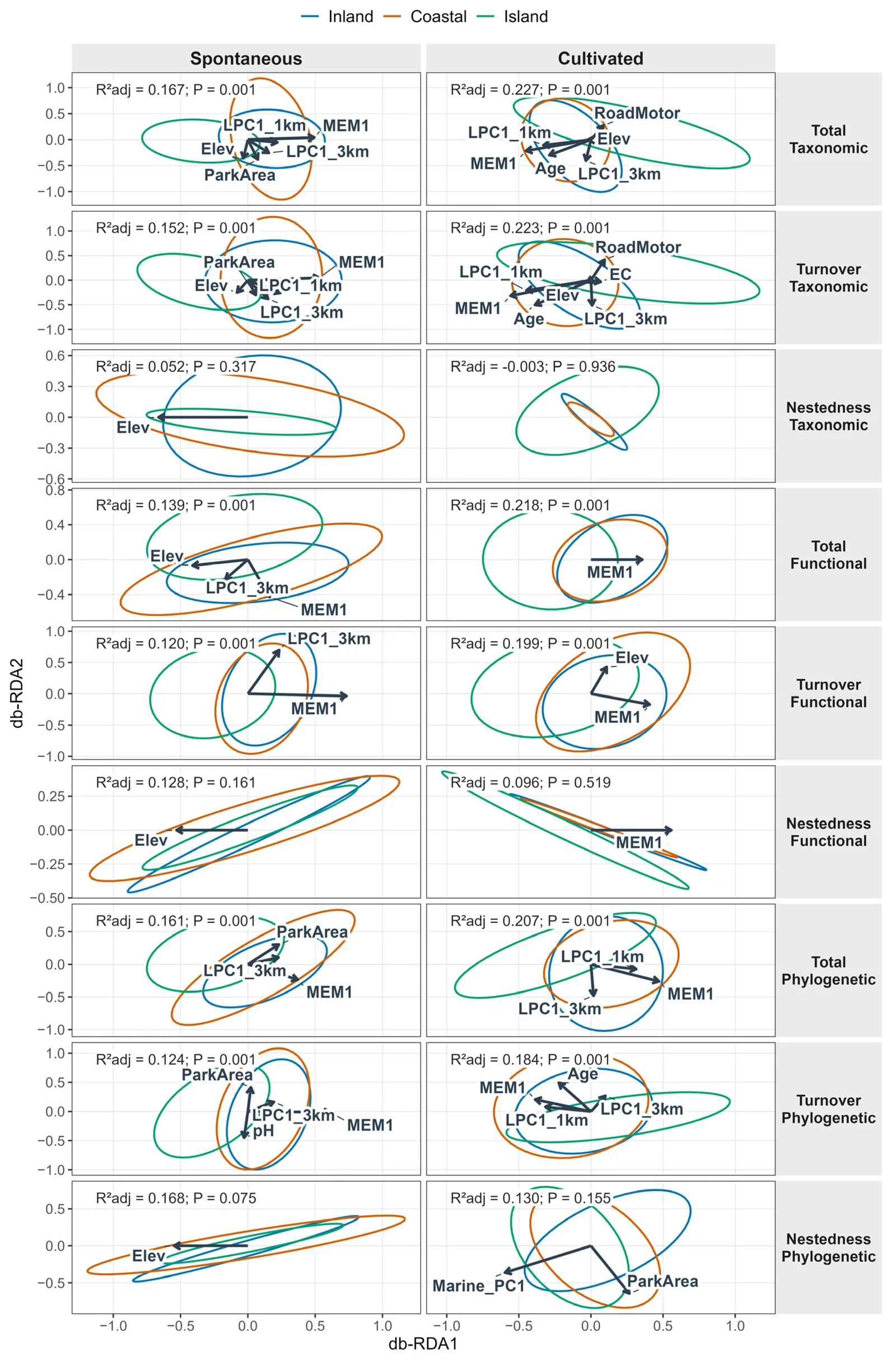
Ordinations of the 18 distance-based redundancy analysis (db-RDA) models and the factors retained by forward selection. Panels are arranged by component (Total, Turnover, Nestedness) and dimension (Taxonomic, Functional, Phylogenetic), with the spontaneous and the cultivated groups on the left and right sides, respectively. Each panel corresponds to one final forward-selection model, and arrows represent the factors retained by that model (the minimal sufficient set of variables identified by forward selection). The R^2^_adj_ and *p* values in the upper left corner of each panel are the fit and permutation test of the full model containing all candidate variables, which is not the same model: the adjusted R^2^ of the full model is systematically higher than the adjusted R^2^ of the retained variable set itself. Ellipses in each panel are 95% confidence ellipses of the three regions on the constrained axes. Abbreviations: MEM1 = spatial eigenvector 1; ParkArea = park area; Elev = elevation; LPC = landscape principal components (LPC1 and LPC2 correspond to PC1 and PC2); Age = park age; RoadMotor = motor access; EC = soil electrical conductivity; Marine_PC1 = the marine stress factor.

**Figure 6:**
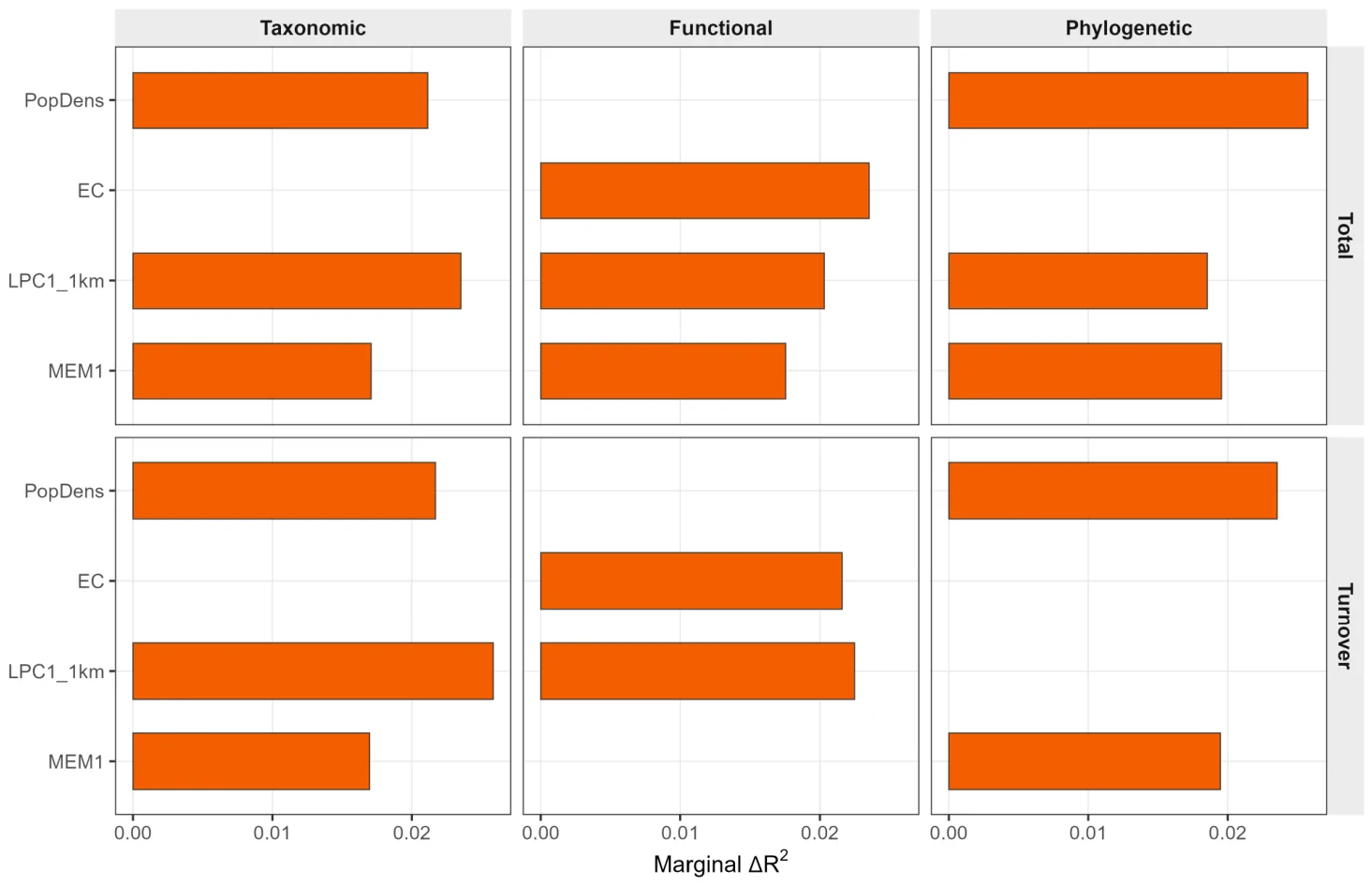
Significant marginal effects in the cultivated group. Marginal effects are the ΔR^2^ of a single factor after accounting for all remaining factors in the full db-RDA model (containing all candidate variables); the factors shown therefore need not appear among the forward-selection arrows in Figure 5. Only factors significant after FDR correction (q < 0.05) are displayed, and all of them belong to the cultivated group.

The db-RDA results (Figure 5) showed that the full models were significant for total beta diversity (R^2^_adj_ = 0.139–0.227, *p* < 0.05) and for the turnover component (R^2^_adj_ = 0.120–0.223, *p* < 0.05), whereas none was significant for the nestedness component (*p* > 0.05). The marginal-effects results (Figure 6) showed that all significant marginal effects were in the cultivated group, involving Landscape PC1 (1 km), population density, the spatial eigenvector MEM1 and soil electrical conductivity; no factor was significant in the spontaneous group. The forward-selection results (Figure 5) showed that the spatial eigenvector MEM1 was retained in 13 of the 18 models; total beta diversity and the turnover component of the cultivated taxonomic and phylogenetic dimensions were explained by factors including Landscape PC1 (3 km), Landscape PC1 (1 km), motor access, park age and soil electrical conductivity, whereas in the spontaneous group, park area and elevation were retained in addition to MEM1.

### 3.4. Regional Differences and Drivers of Plot-Level Local Contributions

**Figure 7:**
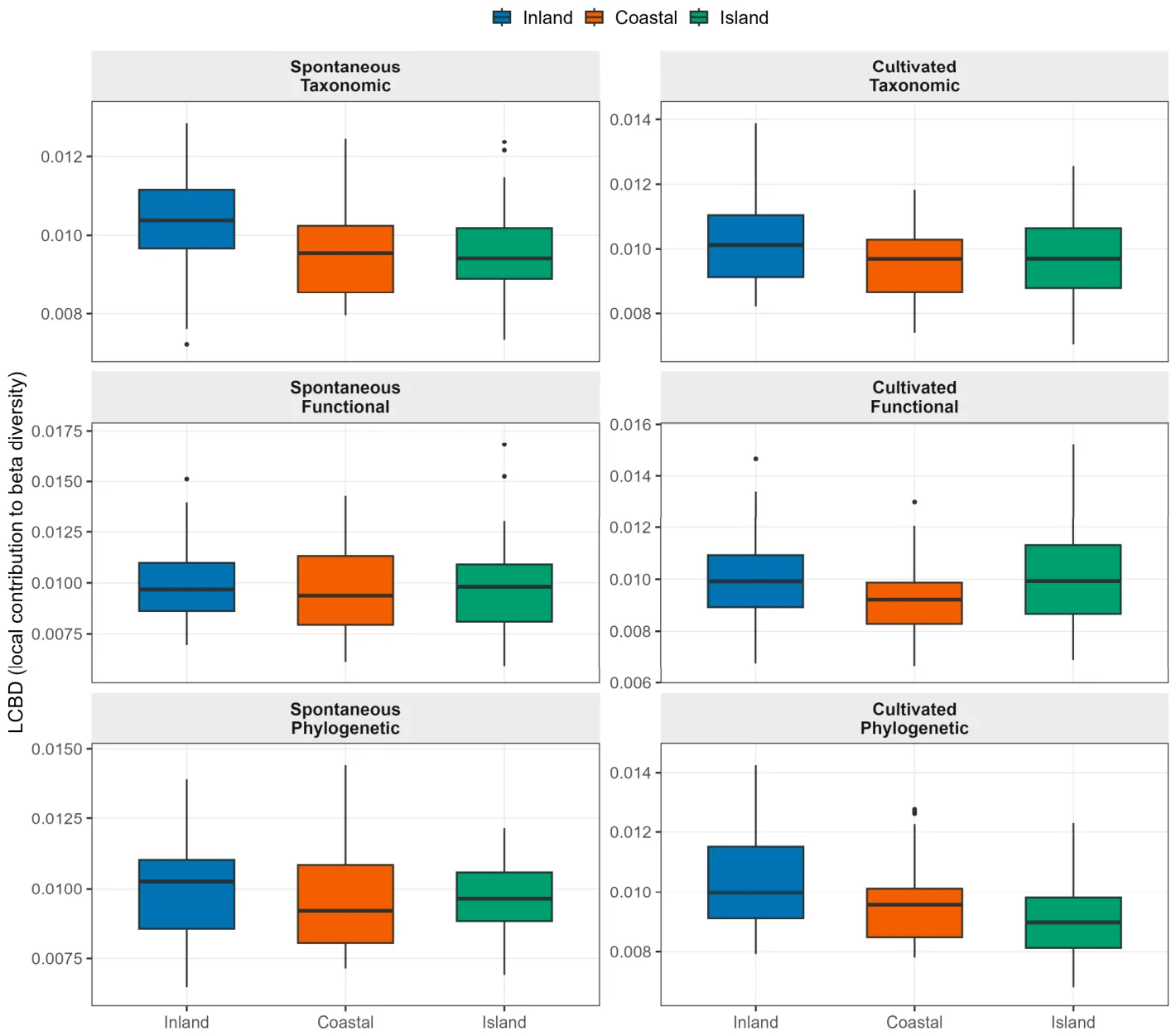
Distribution of the local contribution to beta diversity (LCBD) of plots across the three regions, shown for the taxonomic, functional and phylogenetic dimensions of the spontaneous and cultivated groups. Black points in the panels are outliers (values beyond 1.5 times the interquartile range).

Regional differences in the LCBD were limited (Table S3): among the six responses, only cultivated phylogenetic beta diversity differed among regions, with higher values in the Inland than in the Island region (pairwise comparisons Holm-corrected, *p* < 0.05); the remaining responses were not significant (*p* > 0.05). Permutation tests of the LCBD showed that the contribution of plot MJN2 in the cultivated taxonomic dimension was significantly higher than expected by chance (*p* = 0.01), whereas no plot was significant in the spontaneous group (*p* > 0.05); under the within-park stratified null model, which preserved the species composition of each park, plots MJN2 (cultivated group) and NS7 (spontaneous group) were significant (*p* = 0.01). Across the three regions, the LCBD of taxonomic and phylogenetic beta diversity was highest in the Inland region, with similar values in the Coastal and Island regions; for functional beta diversity, the cultivated group showed similar values in the Inland and Island regions and the lowest values in the Coastal region, whereas the three regions were similar in the spontaneous group (Figure 7). Along the continuous gradients, only the marine stress factor was correlated with the LCBD of the cultivated group (taxonomic, β = −0.044, *p* < 0.05; phylogenetic, β = −0.073, *p* < 0.01); all other gradient variables were non-significant (Table S4). The factors initially retained by stepwise regression (Table S5) were dominated by the marine stress factor, soil temperature, park area, elevation and Landscape PC2 (3 km) in the spontaneous group, and by Landscape PC1 (1 km), Landscape PC1 (3 km), population density, management intensity and soil pH in the cultivated group. The independent contributions from hierarchical partitioning (Table 3) showed that park area ranked first in all three dimensions of the spontaneous group (21.9–57.2%), followed in the taxonomic dimension by elevation and the shape index (16.6% each), whereas Landscape PC1 (3 km) dominated all three dimensions of the cultivated group (50.3–66.0%). The association between the LCBD and plot-level species richness was negative in the spontaneous taxonomic dimension (r = −0.264, *p* < 0.01) and non-significant in all three dimensions of the cultivated group (*p* > 0.05, |r| < 0.19). The top three SCBD species in the spontaneous group were large-flowered beggarticks (*Bidens alba*), Oriental false hawksbeard (*Youngia japonica*) and Southern crabgrass (*Digitaria sanguinalis*), and those in the cultivated group were Japanese lawngrass (*Zoysia japonica*), Chinese banyan (*Ficus microcarpa*) and Bermudagrass (*Cynodon dactylon*); the remaining species are listed in Table S6.

### 3.5. Piecewise Structural Equation Models of Plot-Level Local Contributions

**Figure 8:**
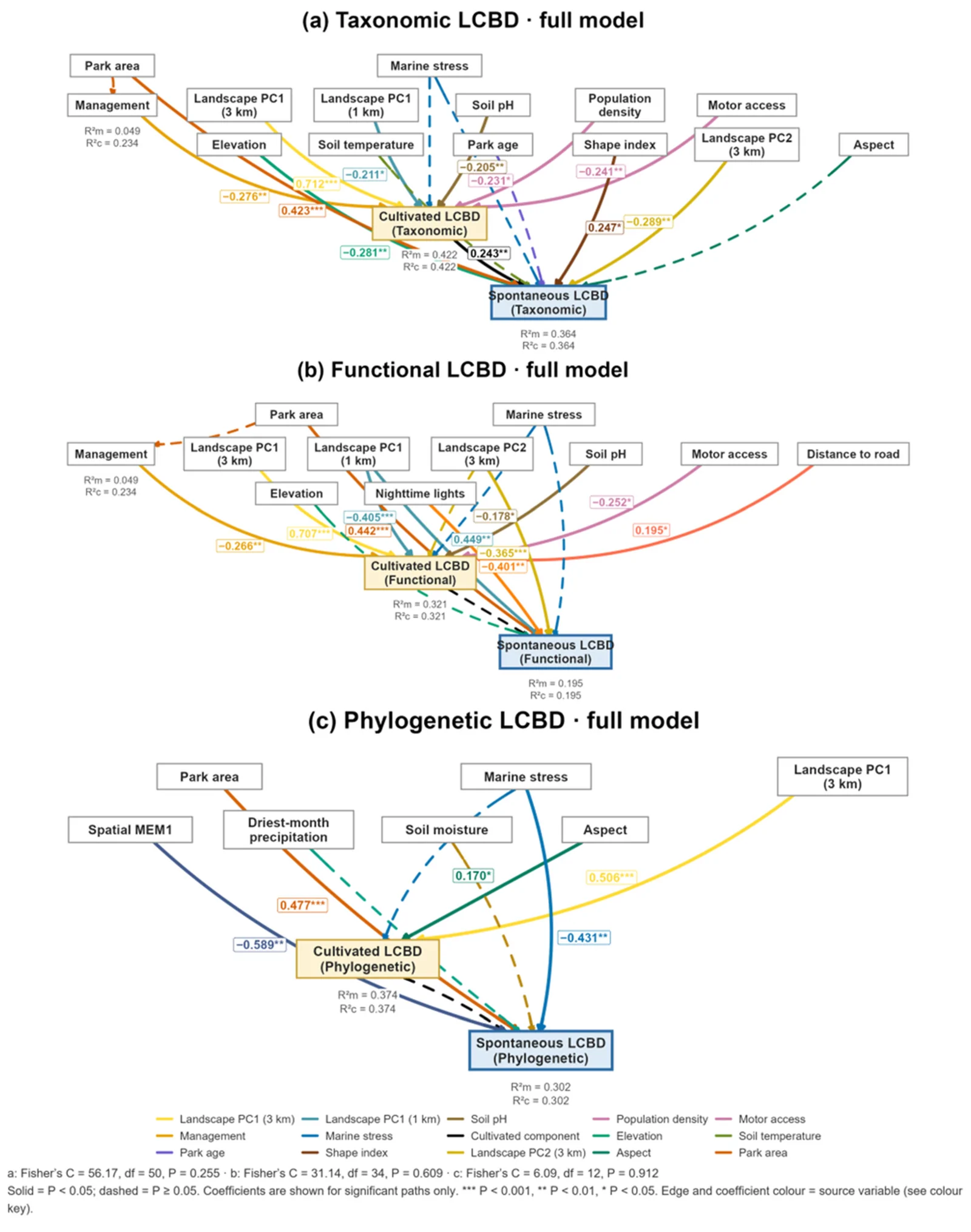
Structural equation model (SEM) pathways of the three local contributions to beta diversity (LCBD) models, shown as three panels: (**a**) taxonomic, (**b**) functional and (**c**) phylogenetic. Solid lines denote pathways with *p* < 0.05 and dashed lines pathways with *p* ≥ 0.05; coefficients are standardized estimates. Asterisks denote significance levels: * *p* < 0.05, ** *p* < 0.01 and *** *p* < 0.001; coefficients are shown for significant paths only. R^2^_m_ and R^2^_c_ in the panels are the marginal (fixed effects only) and conditional (fixed effects plus the random effect of park ID) R^2^ of each equation within the SEM and differ in definition from the R^2^_m_ of the final stepwise-regression models (see Table S5); Fisher’s C test results of the three models are given at the bottom of the figure.

The piecewise SEM results (Figure 8) showed that all three models passed Fisher’s C tests (*p* > 0.05). The cross-group pathway (from the cultivated to the spontaneous group) was significant only for taxonomic beta diversity (β = +0.243, *p* < 0.01) and was not significant for functional or phylogenetic beta diversity (*p* > 0.05). Among the direct effects on the LCBD of the spontaneous group, park area was significantly positive for all three dimensions of beta diversity (β = +0.423, +0.442 and +0.477, *p* < 0.001), and taxonomic beta diversity additionally showed negative effects of Landscape PC2 (3 km) (β = −0.289, *p* < 0.01) and elevation (β = −0.281, *p* < 0.01). Among the direct effects on the LCBD of the cultivated group, Landscape PC1 (3 km) was significantly positive for all three dimensions of beta diversity (β = +0.712, +0.707 and +0.506, *p* < 0.001), whereas management intensity, motor access and population density were negative. The remaining pathways are shown in Figure 8.

## 4. Discussion

### 4.1. Two Kinds of “Turnover”: Planting Decisions and Ecological Replacement

The turnover component corresponds to species replacement among plots and the nestedness component to richness differences (i.e., species loss); the two arise from different processes, and distinguishing them is a prerequisite for inferring causes [16,17]. In this study, beta diversity in both groups was dominated by the turnover component (Table 1), consistent with the general pattern [18].

For spontaneous species, turnover is closer to species replacement in the usual sense: similarity among plots declined with geographic distance (Figure 3), which may arise from environmental differences that vary with distance or from dispersal limitation [56]. Partial Mantel tests showed that, after controlling for spatial distance, the correlation between marine exposure and spontaneous beta diversity was no longer significant, whereas the correlation with spatial distance remained significant after controlling for the non-marine environment (Figure 4). Turnover in the spontaneous group therefore reflects species replacement shaped jointly by environmental and spatial processes, and their relative contributions cannot be separated.

In contrast to the spontaneous group, turnover in the cultivated group is more likely to record differences in planting decisions than species replacement in the ecological sense. The assembly of cultivated species lists approximates a filter of “human preference”: which species are introduced, and where they are planted, is determined mainly by introduction and management [21], and the plant composition of yards is determined by yard area and the characteristics of the surrounding landscape [57]. The turnover proportion was higher in the cultivated group than in the spontaneous group (Table 1), and directional tests supported opposite distance trends in the nestedness component of the two systems: functional and phylogenetic nestedness increased significantly with distance in the cultivated group, whereas nestedness decreased significantly in all three dimensions of the spontaneous group, and the difference in slopes between the two systems was significant in all three dimensions (taxonomic dimension, *p* < 0.01; functional and phylogenetic dimensions, *p* < 0.001; Table S8). This pattern is compatible with the interpretation that cultivated plots farther apart increasingly represent “reduced subsets of the inland flora,” but two limitations apply. First, the nestedness component of beta diversity is a symmetric metric that does not by itself indicate which side is the subset; the direction therefore relies on auxiliary evidence such as dispersion. Second, the pattern appeared in the functional and phylogenetic dimensions, whereas no distance trend was detected in the nestedness component of the cultivated taxonomic dimension (*p* > 0.05); the “reduction of species lists” interpretation therefore applies only at the functional and phylogenetic levels and cannot be extended directly to a strictly nested sequence of taxonomic lists. It is consistent with a planting outcome of “fewer species, with tolerant tree species substituted,” rather than with replacement along an environmental gradient.

The nestedness component was low in both groups (Table 1): differences among plots mainly reflect “species being replaced” rather than “a set of species being lost.” These observations only show that the pattern is compatible with the decision-based explanation; we did not measure planting decisions directly, and differences in the tolerance of cultivated species and in nursery stock supply may also be involved, so the candidate mechanisms cannot be distinguished.

The two kinds of turnover may also be related to plant life strategies [58]. In Grime’s C-S-R framework, competition, stress and disturbance define three primary strategies, and disturbance (such as trampling and mowing) is explicitly recognized as a selective force [58]. The persistence of cultivated species depends on planting, watering and pruning, so their occupancy of a plot reflects the “anthropogenic strategy”—a strategy of human dependence that exists only in maintained urban green space—rather than an autonomous response to habitat conditions. Spontaneous species occupy lawns and edges that are mown, weeded and trampled on municipal schedules: such low-stress, high-disturbance conditions select for ruderals (R), consistent with the dominance of warm-season annual weeds such as *B. alba*, *Y. japonica* and *D. sanguinalis* among the top SCBD contributors (Section 4.4; Table S6). Direct evidence for stress-tolerant (S) and competitive (C) strategies is lacking in this study, but the former may persist on dry, wind-exposed coastal sites and the latter in less disturbed, irrigated patches. The three leaf traits used here (SLA, leaf area and LDMC) are precisely the traits on which the global CSR classification is based [59], so the lower functional than taxonomic beta diversity (Table 1) also implies that replaced species mostly share similar leaf-economic strategies.

### 4.2. Functional and Phylogenetic Redundancy of Taxonomic Replacement

Beta diversity is not measured in a single way, and the selective joint use of multiple measures helps reveal the ecological structure behind it [14]. In this study, beta diversity in all three dimensions increased with distance; taxonomic beta diversity was the highest and showed the largest increase (Table 1 and Table S8, Figure 3), whereas functional and phylogenetic beta diversity were lower. Ecological traits are phylogenetically conserved: closely related species often share similar functional attributes [60], and phylogenetic beta diversity carries information on both environmental filtering and evolutionary history [61]. The lower functional and phylogenetic beta diversity is therefore more likely to reflect trait and phylogenetic similarity among the replaced species than a reorganization of functional configurations; this explanation depends on the resolution of the trait table and the phylogeny and remains suggestive.

Functional redundancy requires species to overlap in both functional effects and environmental tolerance; similar traits do not guarantee compensation at the population level [62,63]. The piecewise SEM showed that the pathway from the LCBD of the cultivated group to that of the spontaneous group was significant only for taxonomic beta diversity (Figure 8): plot-level synchrony between the two groups occurred only in species composition and did not extend to functional or phylogenetic composition. This is consistent with findings from German urban areas [10], where high species richness derives mainly from closely related, functionally similar groups. The non-significant functional and phylogenetic pathways may also reflect the limited precision of the trait and phylogenetic information.

The implication of this pattern is that species richness cannot serve as a proxy for the other dimensions of diversity [11]: if the diversity targets of urban greening rely on species richness as a proxy, changing species or their arrangement does not guarantee a change in the functional configuration of the community.

### 4.3. The Level of Action of Marine Exposure and Divergent Drivers of the Two Plant Groups

The relationship between marine exposure and beta diversity lost significance for most responses once spatial distance was controlled (Figure 4), suggesting that its action may be concentrated at the regional rather than the plot scale. The regional species pool comprises all species able to colonize a target site [64]. Cultivated plants undergo frequent introduction, replacement and maintenance and are particularly subject to species-pool effects; which species enter the pool is constrained by both environmental and anthropogenic filtering [21]. Marine exposure belongs to environmental filtering and restricts the range of available species at the regional scale, whereas beta diversity responds to a factor only when that factor creates compositional contrasts at the plot scale. Marine exposure and spatial location cannot be fully separated, and effects are transmitted through the spatial structure. On the evidence available here, the action of marine exposure is concentrated at the regional scale, filtering the species pool rather than structuring compositional differences among plots. This explanation is a post hoc, exploratory inference: we did not measure the regional species pool (regional species lists or gamma diversity) directly. It should be noted that we treat the variation partitioning and the spatially explicit db-RDA as the primary evidence on marine exposure and regard the partial Mantel results as supportive only, because partialling one distance matrix from another cannot fully separate collinear effects.

At the plot scale, the two groups responded to different categories of drivers. The factors retained for the cultivated group were dominated by Landscape PC1 (1 km), Landscape PC1 (3 km), motor access and park age (Figure 5), and the factors with independent marginal contributions were Landscape PC1 (1 km), population density, the spatial eigenvector MEM1 and soil electrical conductivity (Figure 6). Urban plant composition is significantly correlated with socioeconomic variables [65], and landscape designers are the dominant drivers of cultivated composition [23]. For the spontaneous group, the retained factors were dominated by the spatial eigenvector MEM1, park area and elevation (Figure 5), and no factor had an independent marginal contribution. Spatial eigenvectors are constructed from the spatial relationships among plots [42] and are regarded as proxies for unmeasured processes [43]; the selection of MEM1 indicates that compositional differences in the spontaneous group contain a clear spatial structure, but this cannot be equated directly with dispersal limitation [43]. The role of park area may arise from the habitat conditions that accompany area [66], but whether it operates through habitat heterogeneity, the size of the seed-source pool, or the area effect itself cannot be distinguished.

Beta diversity showed regional differences in all three dimensions of the spontaneous group, and likewise in the cultivated group (Table 2); in the ordination space, the Island-region plots separated clearly for the three cultivated dimensions and for the spontaneous taxonomic dimension (Figure 2). Compositional differences among regions took the form of species replacement: each region holds its own adapted or planted species, and the regional lists interlock rather than forming a nested sequence (Table 1). This interlocking at the level of regional lists does not conflict with the nestedness direction at the plot-pair scale described in Section 4.1: the former states that no strict containment relationship exists among the lists of the three regions, whereas the latter describes how the nestedness component changes with geographic distance. It should be noted that dispersion also differed among regions for two responses, the spontaneous taxonomic and the cultivated phylogenetic (Table 2), so differences in location and in dispersion are difficult to separate completely.

The LCBD apportions regional beta diversity among individual plots and reflects the contribution of each plot to regional differentiation [38]. The driver structures of beta diversity and of the LCBD were inconsistent within both groups: beta diversity was determined mainly by spatial structure and by park location and site conditions, whereas the LCBD was additionally dominated by park area in the spontaneous group (independent contributions of 21.9–57.2%) and by Landscape PC1 (3 km) in the cultivated group (50.3–66.0%) (Table 3, Figure 8); MEM1 was retained only for the phylogenetic dimension of the spontaneous group. Hierarchical partitioning further shows that cultivated beta diversity is more concentrated under the control of a single landscape factor: Landscape PC1 (3 km) ranked first in the taxonomic, functional and phylogenetic dimensions of the cultivated group, whereas the first-ranked factor in all three dimensions of the spontaneous group was park area, with landscape configuration ranking second only in the functional dimension.

### 4.4. Sources of Local Uniqueness and Spatial Homogenization

The SCBD further apportioned regional beta diversity among individual species (Table S6): the highest contributors in the spontaneous group were unevenly distributed weed-like species, whereas those in the cultivated group were lawn grasses and common landscape trees. The LCBD of the spontaneous group was determined by the presence or absence of these weeds, and larger parks had higher LCBD; plot-level species richness was negatively correlated with the LCBD in the spontaneous group, opposite to the direction expected from species–area effects, indicating that the role of park area cannot be explained by differences in species richness [67]. The LCBD of the cultivated group was determined by differences in planting, and intensively managed plots were closer to the standard combination of lawn grasses and landscape trees (Figure 8).

Biotic homogenization refers to the increase in similarity among biota over time; because this study was a single survey, this temporal process cannot be determined [27]. What can be tested is spatial homogenization at a single point in time, that is, differences in the compositional similarity of plots within a region [19,68]. All six responses separated significantly among regions, with the Island-region plots deviating as a whole (Figure 2). Within regions, dispersion differed among regions only for the cultivated phylogenetic and the spontaneous taxonomic responses (Table 2), both showing the highest values in the Inland region, intermediate values in the Coastal region and the lowest values in the Island region; for the remaining four responses, dispersion was also highest in the Inland region but not significantly so. Among these four, only the cultivated functional dimension reversed the coastal–island order, with islands slightly higher than the coast, and in the cultivated taxonomic dimension the two were equal (both 0.537) (Table 2). Differentiation among regions and concentration within regions can occur simultaneously [27]: the former corresponds to differences in marine exposure and urbanization intensity among the three regions, whereas the latter may relate to the relatively uniform conditions within regions. This also suggests that homogenization at the level of urban floras is not a universal outcome of urbanization; its direction depends on the species group and the observational scale [29,30].

Spatial homogenization in the cultivated group appeared in the phylogenetic dimension, where the Island-region plots had the most concentrated composition (Table 2); the phylogenetic LCBD was lower on islands than in the Inland region, and the LCBD of the cultivated group declined with increasing marine stress (Tables S3 and S4). This may reflect a narrowing of planting sources: as marine exposure intensifies, the filtering of species introduction, maintenance and nursery stock supply reduces the phylogenetic combinations available for planting, so the plantings that remain are more similar to one another. Differences in planting may also originate on the supply side: narrower nursery stock sources on islands, higher logistics costs and different municipal procurement channels can all produce the same convergent pattern even in the absence of differences in decision-making; post-planting tolerance filtering (salt spray and wind) likewise screens the species that persist. We did not collect introduction lists, nursery stock sources or planting records, so the three mechanisms (decisions, supply and tolerance filtering) cannot be distinguished; their distinguishable predictions are differences in introduction lists, in nursery stock sources and in post-planting mortality, respectively. Compositional convergence of cultivated plants among cities is usually attributed to the use of a few common species [25], and continental-scale comparisons of residential lawns provide empirical support for this view [36]. Convergence of planting along environmental gradients within a city may arise from the same class of mechanism: the same list of common species is planted repeatedly over a smaller gradient.

Spatial homogenization in the spontaneous group appeared in the taxonomic dimension, where the Island-region plots had the most concentrated composition (Table 2): the differences among plots lie in the replacement of species identities, and the Island-region plots are more similar to one another in this replacement while separating as a whole from the other two regions (Figure 2). This may reflect a narrowing of the available species sources: the range of species able to occur may shrink along the marine exposure gradient, making compositions more similar to one another; however, the spontaneous group lacks corresponding evidence at the plot level (no regional differences were detected in Table S3), so this explanation can only be regarded as suggestive.

The sources of local uniqueness and the causes of spatial homogenization correspond to different management levers. The naturalizing species that carry the LCBD of the spontaneous group, such as B. alba, are both an important source of spontaneous beta diversity and targets of naturalization monitoring: weeding suppresses their occurrence but simultaneously compresses spontaneous beta diversity, so a trade-off exists between the two. This trade-off is partly definitional, since removing high-contributing species necessarily reduces their contribution. The LCBD of the cultivated group is carried by planted species, and planted species can spread and enter the spontaneous state [69]; if cultivated species continue to escape and naturalize, present differences in planting may be transformed into sources of spontaneous beta diversity in the future. Homogenization in the cultivated group is associated with differences in planting, and introduction lists and maintenance standards constitute a testable moderating pathway: if the hypothesis of narrowing planting sources holds, changes in lists and maintenance standards should predictably change the compositional similarity of the cultivated group. However, the authority to revise introduction lists usually lies with municipal horticultural departments rather than individual parks, and centralized procurement, survival-rate acceptance and maintenance assessment all favor a few common species, so the practical magnitude of this lever is institutionally constrained. For the spontaneous group, which is associated with the regional species pool and park area, the levers lie in park size and habitat heterogeneity. In addition, the weeding trade-off described above suggests that zone-based weeding—tolerating high-SCBD species in low-traffic areas while concentrating removal where they conflict with park use—could reconcile the maintenance of spontaneous beta diversity with management needs.

Management also shapes the spontaneous component, but through a different channel. Park soils are imported or amended and moisture is regulated by watering, so the habitats available to spontaneous plants are engineered rather than natural: these modifications raise soil fertility and available moisture and thereby favor nutrient-demanding, disturbance-tolerant annuals, and the site conditions of such novel urban ecosystems differ fundamentally from those of the surrounding landscape [70]. Frequent mowing and weeding on municipal schedules keep these habitats in a recurrently disturbed, early-successional state dominated by herbaceous swards [71], the short mown lawn being the most widespread form of public greenspace in temperate cities [72]. In this study, the spontaneous taxa occurring in most parks were predominantly ruderal, trampling-tolerant and lawn-associated species (Section 3.1): frequent low cutting favors low-growing annual plants and grasses [73], and reducing mowing frequency rapidly increases species richness [74] and taxonomic, functional and phylogenetic diversity [75], indicating that the mowing regime itself filters which spontaneous species persist. In the models, however, management intensity (MF) was retained only for the cultivated group (Table 3; Figure 8): intensively managed plots converged on the standard combination of lawn grasses and landscape trees, whereas spontaneous beta diversity was associated with spatial structure, park area and elevation rather than with MF. Two cautions apply. First, MF captures access and trampling, pruning and clearing, and built facilities rather than a direct census of weeding effort, so the absence of an MF effect in the spontaneous group does not imply that management is irrelevant to spontaneous vegetation. Second, park-level maintenance practices (e.g., weeding operations) may also constrain spontaneous beta diversity indirectly through the trade-off discussed above: suppressing high-SCBD species such as *B. alba* could simultaneously compress spontaneous beta diversity. In sum, the engineered and repeatedly disturbed conditions of parks keep spontaneous vegetation in an early-successional state dominated by ruderal and trampling-tolerant species, and management, through the mowing and weeding regime, shapes the species composition of spontaneous vegetation mainly as a disturbance filter, even though this effect was not captured by the plot-level management intensity score (MF).

### 4.5. Limitations and Outlook

The conclusions of this study are subject to the following limitations. (1) Seed sources, soil seed banks and disturbance history were not recorded, and management entered the models only as the plot-level intensity score (MF), which reflects local within-plot management but not park-level maintenance details and inputs, so planting decisions, nursery stock supply and post-planting tolerance filtering cannot be distinguished as mechanisms; the analyses were based on incidence data and cannot separate replacement at the abundance level from replacement of species identities, because abundance is not comparable between the two groups (see Section 2.2). (2) The nestedness component was low, and its independent effects were not significant in any model (Table 1; Figure 5 and Figure S1), although its directional change with distance was supported by permutation tests (Figure 3). (3) Marine exposure cannot be fully separated from spatial location and region labels, so its independent effect cannot be confirmed (Figure 4); the design is limited to three discrete regions. (4) The SCBD was computed only from incidence data in the taxonomic dimension (Table S6). (5) The conclusions derive from a survey in a single city and a single season; the top three SCBD species (*B. alba*, *Y. japonica* and *D. sanguinalis*) are all warm-season annual weeds, so the results apply to summer communities, and seasonal dynamics may change the ranking of local contributions. The three regions were also surveyed sequentially (Island, then Inland, then Coastal; Table S10), so differences in the composition of annual species among regions may partly reflect survey timing. (6) Species not included amounted to 29.4% and 0.3% in the functional and phylogenetic dimensions of the spontaneous group, respectively, and 12.4% and 4.8% in the cultivated group: in the functional dimension, these were species that still lacked complete values for the three leaf traits after imputation, together with low-frequency species; in the phylogenetic dimension, they were the single spontaneous species without a GBOTB record and, for the cultivated group, the entries removed when cultivars were merged into their parent species (12 entries, whose information still entered the analysis through the tips of the parent species), which is not a coverage gap.

Longitudinal monitoring could determine whether differences in planting converge or diverge and how within-region concentration changes over time; adding qualitative management details, introduction lists and seed sources could test whether planting sources narrow as stress intensifies; updating the LCBD and SCBD on a consistent basis would allow spatial homogenization to be tracked, and the resulting species lists could be linked to weed-management lists.

## 5. Conclusions

Drawing on 102 plots in 21 urban parks along the coastal island-to-mainland gradient in Fuzhou, and separating marine exposure from spatial structure, we decomposed in parallel the taxonomic, functional and phylogenetic beta diversity of cultivated and spontaneous plants into turnover and nestedness components, attributed their driver structures, and apportioned beta diversity to the plot level. The main conclusions are as follows:

(1) Beta diversity in both groups was dominated by the turnover component, but the two kinds of turnover carry different meanings. Turnover in the spontaneous group reflects species replacement shaped jointly by environmental and spatial processes, and its nestedness component decreased significantly with distance; the cultivated group had a higher turnover proportion and a significantly lower nestedness component, and nestedness in its functional and phylogenetic dimensions increased significantly with distance (*p* < 0.01), consistent with different planting processes. The pattern in the cultivated group is compatible with the interpretation that more distant plots represent reduced subsets of the inland species list, but this interpretation is limited to the functional and phylogenetic levels, and differences in the tolerance of cultivated species and in nursery stock supply have not been excluded.
(2) Functional and phylogenetic beta diversity were consistently lower than taxonomic beta diversity, and cross-group synchrony in local contributions occurred only in the taxonomic dimension: species replacement was not accompanied by corresponding functional and phylogenetic differentiation, and species richness cannot serve as a proxy for the other dimensions of diversity.
(3) The driver structures of the two groups diverged. Beta diversity in the cultivated group was dominated by anthropogenic factors such as landscape configuration, population density and motor access, whereas beta diversity in the spontaneous group was associated with spatial structure, park area and elevation. Marine exposure lost significance for most responses once spatial distance was controlled, suggesting that its action may be concentrated at the regional scale, through filtering of the species pool; this inference is post hoc and exploratory, and we did not measure the regional species pool directly.
(4) Composition differentiated significantly among regions, and within-region composition was most concentrated in the Island region, in the phylogenetic dimension of the cultivated group and in the taxonomic dimension of the spontaneous group, suggesting that marine stress may promote spatial homogenization by narrowing planting sources and the range of available species. Local contributions were explained by park area in the spontaneous group and by landscape configuration in the cultivated group, so the management levers differ accordingly: a trade-off exists between weeding and the maintenance of spontaneous beta diversity; the moderating pathway of homogenization in the cultivated group (introduction lists and maintenance standards) is a testable hypothesis whose magnitude is constrained by procurement and maintenance institutions; and if cultivated species continue to escape and naturalize, present differences in planting may also be transformed into sources of spontaneous beta diversity in the future.
(5) In practice, cultivated and spontaneous vegetation should be managed as separate targets: spatial homogenization of the cultivated component can be mitigated by diversifying planting lists and nursery procurement, and spontaneous beta diversity maintained through park size and habitat configuration; because the high-SCBD spontaneous species (e.g., *B. alba*) are often also weeding targets, zone-based weeding offers an option that reconciles the maintenance of spontaneous beta diversity with management needs; LCBD and SCBD can serve as monitoring tools that locate priority plots and species; and the escape risk of cultivated species should be screened at the planting stage. These applications are offered as testable options rather than demonstrated prescriptions, because no management treatments were implemented in this study.

In summary, cultivated and spontaneous vegetation should be incorporated in parallel into the assessment and management of beta diversity in urban parks; only long-term monitoring combined with information on introduction lists and seed sources can further test whether differences in planting converge or diverge and how homogenization develops over time.

## Figures and Tables

**Table 1:** Decomposition of beta diversity.

Plant Group	Dimension	Total Beta Diversity	Turnover Component	Nestedness Component	Turnover Proportion
Spontaneous	Taxonomic	0.837	0.744	0.093	0.889
Spontaneous	Functional	0.488	0.320	0.169	0.655
Spontaneous	Phylogenetic	0.519	0.353	0.167	0.679
Cultivated	Taxonomic	0.825	0.767	0.059	0.929
Cultivated	Functional	0.491	0.381	0.110	0.776
Cultivated	Phylogenetic	0.541	0.435	0.106	0.805
Difference (spontaneous–cultivated)	Taxonomic	+0.012 (*p* = 0.001)	−0.022 (*p* = 0.001)	+0.034 (*p* = 0.001)	—
Difference (spontaneous–cultivated)	Functional	−0.003 (*p* = 0.182)	−0.061 (*p* = 0.001)	+0.059 (*p* = 0.001)	—
Difference (spontaneous–cultivated)	Phylogenetic	−0.021 (*p* = 0.001)	−0.082 (*p* = 0.001)	+0.061 (*p* = 0.001)	—

Note: Total beta diversity is the mean pairwise dissimilarity; the *p* values of the differences are permutation *p* values from paired sign-flip permutation tests. Differences, turnover proportions and the relationship βsor = βsim + βsne in this table were computed from unrounded values and may therefore differ by ±0.001 from values obtained by subtracting the three-decimal numbers shown. For the difference rows, the turnover proportion is not applicable (—).

**Table 2:** Permutational multivariate analysis of variance (PERMANOVA) and tests of the homogeneity of multivariate dispersions among the three regions.

Plant Group	Dimension	PERMANOVA F	*p*	Dispersion F	*p*	Mean Dispersion (Inland/Coastal/Island)
Spontaneous	Taxonomic	5.269	0.001	6.403	0.040	0.596/0.557/0.523
Spontaneous	Functional	3.671	0.006	0.237	0.854	0.339/0.327/0.327
Spontaneous	Phylogenetic	4.805	0.001	2.236	0.401	0.368/0.349/0.330
Cultivated	Taxonomic	5.177	0.001	4.089	0.173	0.585/0.537/0.537
Cultivated	Functional	6.107	0.001	1.894	0.441	0.342/0.313/0.328
Cultivated	Phylogenetic	5.489	0.002	8.342	0.023	0.394/0.361/0.328

Note: Dispersion is the mean distance of the plots of a region to their group centroid (betadisper; Sørensen dissimilarity; incidence data); smaller values indicate that plot composition is more concentrated in that region. F and *p* values are from tests permuting whole parks as units (21 parks; 999 permutations).

**Table 3:** Independent contributions (%) of the main factors from hierarchical partitioning of the local contribution to beta diversity (LCBD) of plots.

Plant Group	Dimension	Independent Contributions of the Main Factors (%)
Spontaneous	Taxonomic	park area 21.9; elevation 16.6; shape index 16.6
Spontaneous	Functional	park area 35.6; Landscape PC2 (3 km) 28.3; Landscape PC1 (1 km) 19.2
Spontaneous	Phylogenetic	park area 57.2; the marine stress factor 21.7; spatial eigenvector MEM1 9.1
Cultivated	Taxonomic	Landscape PC1 (3 km) 53.0; the marine stress factor 12.1; management intensity 10.6
Cultivated	Functional	Landscape PC1 (3 km) 50.3; Landscape PC1 (1 km) 15.4; management intensity 10.9
Cultivated	Phylogenetic	Landscape PC1 (3 km) 66.0; the marine stress factor 28.6; aspect 5.4

Note: Values are individual contributions (%) from hierarchical partitioning computed by glmm.hp; they are not the same quantity as the unique fractions in the underlying analysis output (the “Unique” column of the corresponding CSV). Only the top three factors are listed in each row; the complete factor list is given in Table S7.

## Data Availability

The data presented in this study are available on request from the corresponding authors due to the ongoing nature of the research project.

## Supplementary Materials

The following supporting information can be downloaded at: https://www.mdpi.com/article/10.3390/biology15211946/s1, Figure S1: Variation partitioning of environmental and spatial effects; Table S1: Full definitions of the environmental factor candidate pool and the modeling variables; Table S2: Taxa overview of the 21 parks (numbers of plots, cultivated and spontaneous taxa per park, and park-level mean management intensity); Table S3: Tests of regional differences in the local contributions of plots; Table S4: Continuous-gradient tests of the local contributions of plots; Table S5: Factor screening for the local contributions of plots; Table S6: Top five species contributions to beta diversity; Table S7: Complete factor list of hierarchical partitioning; Table S8: Distance-decay slopes and permutation tests; Table S9: Software and R package versions; Table S10: Coordinates and survey dates of the 21 parks. The reference cited in the Supplementary Materials [50] also appears in the reference list of the main text.
