# Supplementary material for: Two Kinds of Turnover: Contrasting Drivers of Beta Diversity Between Cultivated and Spontaneous Plants Along a Coastal Island-to-Mainland Gradient
Source: Biology (Basel). 2026 Oct 28;15(21):1946. doi: 10.3390/biology15211946 (PMC13761606; doi:10.3390/biology15211946)
Supplement: Supplementary file 1 [file biology-15-01946-s001.zip › biology-4635030-supplementary.pdf]

# Supplementary Materials

**Figure S1.** Variation partitioning of environmental and spatial effects on beta diversity and its components.

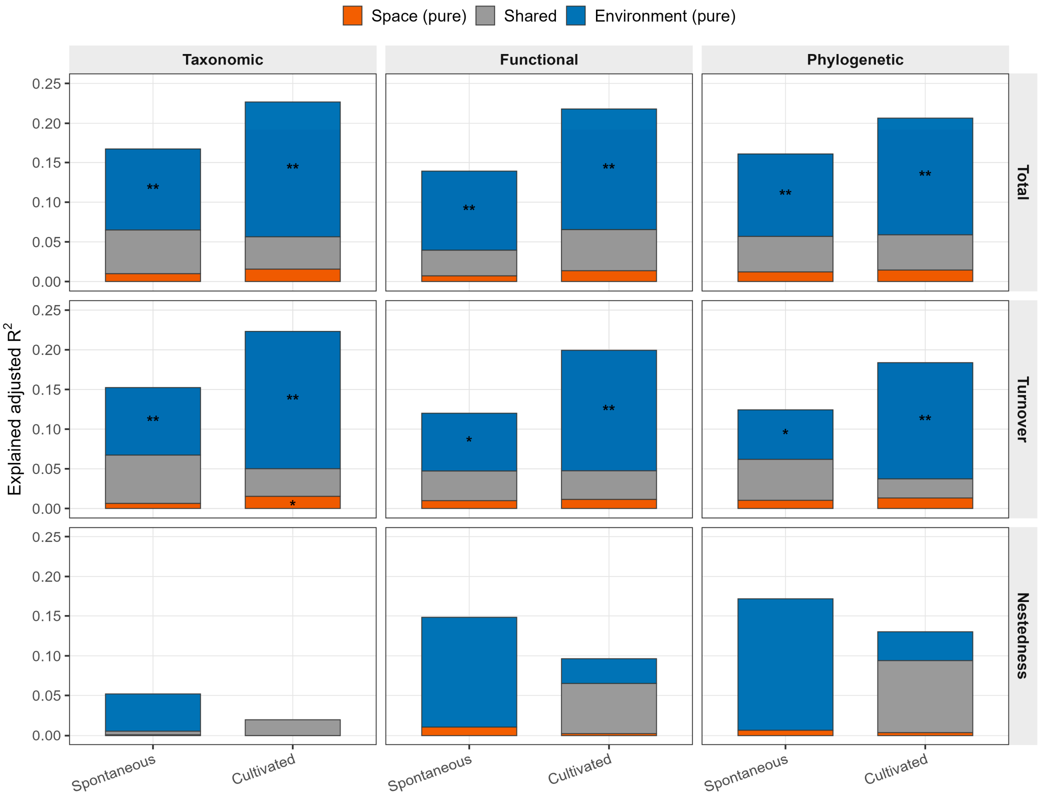


The variation partitioning results (Figure S1) showed that pure environmental effects were significant for total beta diversity and for the turnover component ($R^2_{adj}$ = 0.062–0.173; partial redundancy analysis (partial RDA) permutation tests, P < 0.05), and that the pure spatial effect was significant only for the turnover component of cultivated taxonomic beta diversity ($R^2_{adj}$ = 0.015; partial RDA permutation tests, P < 0.05); the remaining effects were not significant. For total beta diversity, pure environmental effects were higher in the cultivated group ($R^2_{adj}$ = 0.148–0.170) than in the spontaneous group ($R^2_{adj}$ = 0.100–0.104). Neither the pure environmental nor the pure spatial effect was significant for the nestedness component. The unexplained fractions of the 18 responses were above 0.773; for the nestedness component of cultivated taxonomic beta diversity, the adjusted $R^2$ values of the pure environmental, shared and pure spatial fractions summed to a negative value (−0.003), and the unexplained fraction of this response slightly exceeded 1 (1.003).

Note: negative adjusted $R^2$ values were truncated to zero, and the stacked bar heights show sums of these truncated fractions; they should not, therefore, be interpreted as the adjusted $R^2$ of the full db-RDA models (Figure 5 of the main text). The vertical axis shows only the explained fractions (environmental + shared + spatial); the unexplained fractions, all above 0.773, are not plotted. ***, ** and * denote the permutation-test significance of the pure environmental and pure spatial effects at $P < 0.001$, $P < 0.01$ and $P < 0.05$, respectively; the shared fraction was not tested. Negative adjusted $R^2$ values were truncated to 0, so bar heights are sums of truncated overlapping fractions and do not equal the adjusted $R^2$ of the full distance-based redundancy analysis (db-RDA) models: the bar heights for functional nestedness in the spontaneous group and taxonomic nestedness in the cultivated group are approximately 0.149

and 0.020, whereas the $R^2_{adj}$ values of these two responses in Figure 5 of the main text are 0.128 and −0.003; the differences are the sums of the truncated negative fractions. For the nestedness component of cultivated taxonomic beta diversity, the adjusted $R^2$ values of the pure environmental, shared and pure spatial fractions sum to a negative value (−0.003), and the unexplained fraction of this response slightly exceeds 1 (1.003).

**Table S1.** Full definitions of the environmental factor candidate pool and the modeling variables.

| Category | Variable (analysis code) | Unit | Level | Type | Mean ± SD | Range | Data source and processing |
|---|---|---|---|---|---|---|---|
| Management intensity | Management intensity index (MF) | dimensionless (PCA score) | Plot | Continuous | 0.00 ± 1.30 | −2.50 to 2.91 | First PCA axis of three items (trampling, pruning and proximity to facilities), explaining 55.9% |
| Topography | Slope (Slope) | ° | Plot | Continuous | 8.98 ± 6.74 | 0.4–35.0 | Derived from a digital elevation model (DEM) |
| Topography | Elevation (Elev) | m | Plot | Continuous | 29.96 ± 26.37 | −7.9 to 140.6 | Derived from a DEM |
| Topography | Aspect class (Aspect8) | class 1–8 | Plot | Ordinal | 4.04 ± 2.31 | 1–8 | Mapped from azimuth; higher values indicate more sun-exposed aspects |
| Soil | Soil pH (pH) | dimensionless | Plot | Continuous | 5.92 ± 0.61 | 4.10–7.00 | Field measurement; missing values imputed with missForest |
| Soil | Soil water content (SWC) | instrument scale 1–8 | Plot | Continuous | 5.66 ± 1.76 | 1–8 | Field measurement; scale setting 1 corresponds to 10% and setting 8 to 80% volumetric water content; missing values imputed with missForest |

| Category | Variable (analysis code) | Unit | Level | Type | Mean ± SD | Range | Data source and processing |
|---|---|---|---|---|---|---|---|
| Soil | Soil electrical conductivity (EC) | mS/cm | Plot | Continuous | 0.102 ± 0.115 | 0–0.48 | Field measurement; interpreted as a soil fertility indicator, not as a proxy for marine salinity |
| Soil | Corrected soil temperature (ST_corr) | °C | Plot | Continuous | 27.23 ± 2.05 | 22.92–33.28 | Field measurement, corrected to June climate conditions using the air temperature on the measurement day |
| Canopy | Canopy closure (CD) | % | Plot | Continuous | 57.06 ± 22.08 | 15–90 | Visual estimate at the plot center; used only in models of the spontaneous group |
| Canopy | NDVI mean (1 km) | dimensionless | Park | Continuous | 0.272 ± 0.089 | 0.121–0.535 | Mean within a 1-km buffer of the remote-sensing product; used only in models of the spontaneous group |
| Climate | Precipitation of the driest month (BIO14) | mm | Park | Continuous | 33.76 ± 2.40 | 28–37 | WorldClim 2.1 [50] |
| Marine | Marine stress factor (Marine_PC1) | dimensionless (PCA score) | Park | Continuous | 0.00 ± 3.89 | −6.95 to 3.71 | First PCA axis of 16 marine variables (seven salt-spray aerosol, seven wind-field and two sea-breeze-frequency variables; MERRA-2), explaining 93.4%; higher values |

https://doi.org/10.3390/biology15211946

| Category | Variable (analysis code) | Unit | Level | Type | Mean ± SD | Range | Data source and processing |
|---|---|---|---|---|---|---|---|
| | | | | | | | indicate stronger exposure |
| Marine | Sea-facing index (SeaAspect) | −1 to 1 | Park | Continuous | −0.02 ± 0.79 | −1.00 to 0.98 | cos(θ − 158.18°); 1 = directly facing the sea, −1 = directly facing away from the sea |
| Landscape configuration | Landscape PC1 (1 km) | dimensionless (PCA score) | Plot | Continuous | 0.00 ± 3.57 | −8.68 to 7.87 | First PCA axis of 26 landscape-configuration metrics, explaining 48.5% |
| Landscape configuration | Landscape PC1 (3 km) | dimensionless (PCA score) | Park | Continuous | 0.00 ± 3.45 | −7.96 to 10.19 | First PCA axis of 27 landscape-configuration metrics, explaining 43.6% |
| Landscape configuration | Landscape PC2 (3 km) | dimensionless (PCA score) | Park | Continuous | 0.00 ± 2.35 | −6.19 to 3.48 | Second axis of the same PCA, explaining 20.3% |
| Socioeconomic | Nighttime lights (NTL, 1 km) | $nW·cm^{-2}·sr^{-1}$ | Park | Continuous | 24.19 ± 11.37 | 4.89–57.63 | Mean of all monthly composites from 2014 to 2023 of the VIIRS stray-light-corrected product (NOAA/VIIRS/DNB/MONTHLY_V1/VCMSLCFG), averaged within a 1-km buffer |
| Socioeconomic | Population density | persons/km² | Plot | Continuous | 6258 ± 5197 | 82–22,029 | 2020 population raster |
| Socioeconomic | Distance to road | m | Plot | Continuous | 35.9 ± 48.2 | 0.3–241.9 | Nearest distance to road vectors |

| Category | Variable (analysis code) | Unit | Level | Type | Mean ± SD | Range | Data source and processing |
|---|---|---|---|---|---|---|---|
| Socioeconomic | Motor access (RoadMotor) | 0/1 | Plot | Binary | 0.58 (proportion accessible) | 0–1 | Binary flag of road accessibility |
| Park attributes | Park area | ha | Park | Continuous | 31.81 ± 52.76 | 2.37–219.42 | Park boundary vectors |
| Park attributes | Shape index (SI) | dimensionless | Park | Continuous | 1.91 ± 1.43 | 1.07–6.60 | Shape index computed from the perimeter-to-area ratio |
| Park attributes | Park age | years | Park | Continuous | 13.91 ± 10.14 | 1–41 | From the year of establishment to the year of survey |

Note: The candidate pool contains 23 factors in nine categories; after removing canopy closure and NDVI, 21 factors remain for the cultivated group. A factor was assigned to the park level when the ratio of its within-park standard deviation to its total standard deviation was below 0.15 (whole parks were permuted in permutation tests); all other factors were assigned to the plot level (free permutation). Continuous variables are reported as mean ± SD and range (n = 102). PCA scores are standardized principal components with a mean of 0 and no physical units. NDVI = normalized difference vegetation index; NTL = nighttime lights; SI = shape index.

**Table S2. Taxa overview of the 21 parks.**

| Region | Park | Plots | Spontaneous taxa | Cultivated taxa | MF (park mean) |
|---|---|---|---|---|---|
| Inland | Sanjiangkou Ecological Park | 6 | 58 | 43 | −0.62 |
| Inland | Dongjiangbin Park | 8 | 54 | 67 | −0.42 |
| Inland | Yushan Park | 4 | 33 | 59 | 1.41 |
| Inland | Huaqiao Park | 4 | 54 | 38 | 1.36 |
| Inland | Xihe Smart Sports Park | 4 | 32 | 49 | −1.03 |
| Inland | Jinniushan Park | 4 | 56 | 46 | −0.01 |
| Inland | Minjiang Park South Garden | 4 | 60 | 45 | −1.27 |
| Coastal | Dongdaihu Park | 4 | 35 | 26 | 0.77 |
| Coastal | Bingxin Park | 4 | 30 | 34 | 0.62 |
| Coastal | Nanshan Park | 7 | 104 | 70 | 1.46 |
| Coastal | Jiangjunshan Park | 6 | 62 | 31 | 0.75 |
| Coastal | Binhai New Town Sports Park | 5 | 18 | 37 | 0.37 |
| Coastal | Zhenghe Park | 4 | 20 | 18 | −0.28 |

| | | | | | |
|---|---|---|---|---|---|
| Coastal | Changle People's Park | 4 | 23 | 26 | −0.44 |
| Island | 70 Nautical Miles Railway Coast Park | 5 | 36 | 19 | −0.29 |
| Island | Wanbao Park | 4 | 28 | 22 | −1.08 |
| Island | Ruyihu Park | 5 | 33 | 27 | −0.23 |
| Island | Cuiyuan Park | 4 | 28 | 18 | 0.28 |
| Island | Lianhuashan Park | 6 | 68 | 31 | −0.32 |
| Island | Sculpture Garden | 5 | 23 | 27 | −0.69 |
| Island | Longwangtou Ocean Park | 5 | 25 | 9 | −0.57 |

*Note: Taxa counts are the numbers of taxa recorded in at least one plot of the park. Plot counts sum to 34 per region and 102 in total. MF is the park-level mean of the plot-level management intensity index (Section 2.4). Park names and codes follow Table S10.*

**Table S3.** Tests of regional differences in the local contributions of plots.

| Group | Dimension | Region F | P | Marginal $R^2$ | Conditional $R^2$ |
|---|---|---|---|---|---|
| Spontaneous | Taxonomic | 1.651 | 0.220 | 0.062 | 0.305 |
| Cultivated | Taxonomic | 1.910 | 0.178 | 0.090 | 0.468 |
| Spontaneous | Functional | 0.398 | 0.677 | 0.013 | 0.174 |
| Cultivated | Functional | 1.657 | 0.218 | 0.064 | 0.320 |
| Spontaneous | Phylogenetic | 1.011 | 0.382 | 0.038 | 0.277 |
| Cultivated | Phylogenetic | 4.009 | 0.036 | 0.150 | 0.412 |

**Table S4.** Continuous-gradient tests of the local contributions of plots.

| Group | Dimension | Distance to coastline β (P) | Marine stress factor β (P) | Sea-facing index β (P) |
|---|---|---|---|---|
| Spontaneous | Taxonomic | +0.013 (0.506) | −0.035 (0.051) | −0.012 (0.541) |
| Cultivated | Taxonomic | +0.012 (0.588) | −0.044 (0.037) | −0.019 (0.400) |
| Spontaneous | Functional | −0.003 (0.906) | −0.015 (0.595) | −0.022 (0.440) |
| Cultivated | Functional | −0.005 (0.831) | −0.032 (0.199) | −0.032 (0.223) |
| Spontaneous | Phylogenetic | +0.014 (0.576) | −0.034 (0.161) | −0.021 (0.399) |
| Cultivated | Phylogenetic | +0.048 (0.061) | −0.073 (0.002) | +0.003 (0.923) |

Note: β is the standardized coefficient, with the P value in parentheses; each gradient variable was tested against the six responses, giving 18 tests in total.

**Table S5.** Factor screening for the local contributions of plots.

| Group | Dimension | Factors retained (P) | $R^2m$ |
|---|---|---|---|
| Spontaneous | Taxonomic | Marine_PC1 (0.477); ST_corr (0.008); ParkArea (< 0.001); Elev (0.008); LPC2_3km (0.014); Age (0.043); SI (0.043); Aspect8 (0.116) | 0.321 |
| Cultivated | Taxonomic | Marine_PC1 (0.439); LPC1_3km (< 0.001); PopDens (0.016); MF (0.001); pH (0.010); RoadMotor (0.004); LPC1_1km (0.036) | 0.422 |

| Group | Dimension | Factors retained (P) | R²m |
|---|---|---|---|
| Spontaneous | Functional | Marine_PC1 (0.926); ParkArea (< 0.001); LPC2_3km (0.001); LPC1_1km (0.002); NTL_1km (0.005); Elev (0.073) | 0.194 |
| Cultivated | Functional | Marine_PC1 (0.116); LPC1_3km (< 0.001); LPC1_1km (< 0.001); MF (0.004); RoadMotor (0.010); pH (0.041); DistRoad (0.048); LPC2_3km (0.093) | 0.321 |
| Spontaneous | Phylogenetic | Marine_PC1 (< 0.001); ParkArea (< 0.001); MEM1 (0.001); BIO14 (0.021); SWC (0.134) | 0.296 |
| Cultivated | Phylogenetic | Marine_PC1 (0.076); LPC1_3km (< 0.001); Aspect8 (0.037) | 0.374 |

Note: Marine_PC1 = the marine stress factor; ParkArea = park area; Elev = elevation; LPC = landscape principal components (LPC1 and LPC2 correspond to PC1 and PC2; the suffixes 1km and 3km indicate the computation scale); Age = park age; RoadMotor = motor access; EC = soil electrical conductivity; PopDens = population density; MF = management intensity; ST_corr = soil temperature; SI = shape index; NTL_1km = nighttime lights; DistRoad = distance to road; BIO14 = precipitation of the driest month; SWC = soil water content; MEM1 = spatial eigenvector 1; Aspect8 = aspect.

**Table S6.** Top five species contributions to beta diversity.

| Group | Rank | Species | Latin name | Contribution | Frequency of occurrence |
|---|---|---|---|---|---|
| Spontaneous | 1 | Large-flowered beggarticks | *Bidens alba* | 0.0267 | 0.520 |
| Spontaneous | 2 | Oriental false hawksbeard | *Youngia japonica* | 0.0255 | 0.471 |
| Spontaneous | 3 | Southern crabgrass | *Digitaria sanguinalis* | 0.0243 | 0.402 |
| Spontaneous | 4 | Purple nutsedge | *Cyperus rotundus* | 0.0243 | 0.363 |
| Spontaneous | 5 | Chinese hackberry | *Celtis sinensis* | 0.0235 | 0.353 |
| Cultivated | 1 | Japanese lawngrass | *Zoysia japonica* | 0.0284 | 0.500 |
| Cultivated | 2 | Chinese banyan | *Ficus microcarpa* | 0.0284 | 0.500 |
| Cultivated | 3 | Bermudagrass | *Cynodon dactylon* | 0.0279 | 0.588 |
| Cultivated | 4 | Mexican petunia | *Ruellia simplex* | 0.0257 | 0.343 |
| Cultivated | 5 | Chinese fringe-flower | *Loropetalum chinense* var. rubrum | 0.0246 | 0.363 |

Note: Contribution is the per-species additive share of total beta diversity (Sørensen dissimilarity), i.e., the species contribution to beta diversity (SCBD); incidence is the proportion of plots in which the species occurred.

**Table S7.** Complete factor list of independent contributions (%) from hierarchical partitioning of the local contributions of plots.

| Group | Dimension | Independent contributions of factors (%) |
|---|---|---|
| Spontaneous | Taxonomic | park area 21.9; elevation 16.6; shape index 16.6; Landscape PC2 (3 km) 16.2; soil temperature 12.1; the marine stress factor 11.1; park age 3.0; aspect 2.4 |

https://doi.org/10.3390/biology15211946

| Group | Dimension | Independent contributions of factors (%) |
|---|---|---|
| Spontaneous | Functional | park area 35.6; Landscape PC2 (3 km) 28.3; Landscape PC1 (1 km) 19.2; nighttime lights 12.0; elevation 3.5; the marine stress factor 1.3 |
| Spontaneous | Phylogenetic | park area 57.2; the marine stress factor 21.7; spatial eigenvector MEM1 9.1; precipitation of the driest month 6.1; soil water content 6.0 |
| Cultivated | Taxonomic | Landscape PC1 (3 km) 53.0; the marine stress factor 12.1; management intensity 10.6; soil pH 7.6; population density 6.6; Landscape PC1 (1 km) 5.2; motor access 4.8 |
| Cultivated | Functional | Landscape PC1 (3 km) 50.3; Landscape PC1 (1 km) 15.4; management intensity 10.9; soil pH 6.5; the marine stress factor 5.1; motor access 4.5; Landscape PC2 (3 km) 3.9; distance to road 3.3 |
| Cultivated | Phylogenetic | Landscape PC1 (3 km) 66.0; the marine stress factor 28.6; aspect 5.4 |

Note: Values are individual contributions (%) from hierarchical partitioning computed by glmm.hp; they are not the same quantity as the unique fractions in the underlying analysis output (the "Unique" column of the corresponding CSV).

**Table S8.** Distance-decay slopes and permutation tests.

The slope b is the linear-regression coefficient of beta diversity (pairwise dissimilarity) against geographic distance (per 100 km); b > 0 indicates that the component increases with distance and b < 0 that it decreases with distance. P values are from whole-park permutation tests (999 permutations); $R^2$ is the coefficient of determination of the linear fit. The sample comprises all plot pairs among the 102 plots.

| Group | Dimension | Component | b (per 100 km) | 95% CI | $R^2$ | P |
|---|---|---|---|---|---|---|
| Spontaneous | Taxonomic | Total beta | 0.103 | 0.092–0.115 | 0.056 | < 0.01 |
| Spontaneous | Taxonomic | Turnover | 0.182 | 0.164–0.200 | 0.070 | < 0.001 |
| Spontaneous | Taxonomic | Nestedness | −0.078 | −0.090 to −0.066 | 0.030 | < 0.01 |
| Spontaneous | Functional | Total beta | 0.048 | 0.037–0.059 | 0.015 | < 0.01 |
| Spontaneous | Functional | Turnover | 0.096 | 0.083–0.109 | 0.040 | < 0.001 |
| Spontaneous | Functional | Nestedness | −0.048 | −0.062 to −0.034 | 0.009 | < 0.05 |
| Spontaneous | Phylogenetic | Total beta | 0.062 | 0.052–0.073 | 0.026 | < 0.01 |
| Spontaneous | Phylogenetic | Turnover | 0.114 | 0.101–0.127 | 0.053 | < 0.001 |
| Spontaneous | Phylogenetic | Nestedness | −0.051 | −0.065 to −0.038 | 0.011 | < 0.05 |
| Cultivated | Taxonomic | Total beta | 0.109 | 0.097–0.121 | 0.056 | < 0.01 |
| Cultivated | Taxonomic | Turnover | 0.113 | 0.096–0.129 | 0.035 | < 0.01 |

| Group | Dimension | Component | b (per 100 km) | 95% CI | $R^2$ | P |
|---|---|---|---|---|---|---|
| Cultivated | Taxonomic | Nestedness | −0.004 | −0.011 to 0.003 | < 0.001 | > 0.05 |
| Cultivated | Functional | Total beta | 0.091 | 0.081–0.100 | 0.062 | < 0.001 |
| Cultivated | Functional | Turnover | 0.048 | 0.036–0.060 | 0.012 | < 0.05 |
| Cultivated | Functional | Nestedness | 0.043 | 0.032–0.053 | 0.012 | < 0.01 |
| Cultivated | Phylogenetic | Total beta | 0.072 | 0.062–0.082 | 0.036 | < 0.01 |
| Cultivated | Phylogenetic | Turnover | 0.014 | 0.002–0.026 | 0.001 | > 0.05 |
| Cultivated | Phylogenetic | Nestedness | 0.058 | 0.049–0.066 | 0.034 | < 0.001 |

Note: The 95% CIs are least-squares approximations (plot pairs are not independent, so the intervals are narrow); inference is based on the permutation P values.

Differences in slopes between the two systems (spontaneous − cultivated; 999 paired permutations).

| Dimension | Component | Δb | P |
|---|---|---|---|
| Taxonomic | Total beta | −0.005 | > 0.05 |
| Taxonomic | Turnover | +0.069 | < 0.01 |
| Taxonomic | Nestedness | −0.074 | < 0.01 |
| Functional | Total beta | −0.043 | < 0.001 |
| Functional | Turnover | +0.048 | < 0.001 |
| Functional | Nestedness | −0.090 | < 0.001 |
| Phylogenetic | Total beta | −0.009 | > 0.05 |
| Phylogenetic | Turnover | +0.100 | < 0.001 |
| Phylogenetic | Nestedness | −0.109 | < 0.001 |

Note: In the paired permutation, the beta diversity values of the two systems were randomly exchanged within each plot pair, preserving the pairing structure. The differences in nestedness slopes were significant in all three dimensions, so the opposite directions were supported by test.

**Table S9.** Software and R package versions.

| Software/package | Version |
|---|---|
| R | 4.4.3 |
| betapart | 1.6.1 |
| adespatial | 0.3.29 |
| vegan | 2.7.2 |
| lme4 | 1.1.37 |
| lmerTest | 3.1.3 |
| piecewiseSEM | 2.3.0.1 |
| glmm.hp | 1.0.0 |
| MuMIn | 1.48.11 |
| ape | 5.8.1 |
| cluster | 2.1.8.1 |
| V.PhyloMaker2 | 0.1.0 |
| spdep | 1.4.2 |
| permute | 0.9.8 |

https://doi.org/10.3390/biology15211946

| Software/package | Version |
|---|---|
| missForest | 1.6.1 |

**Table S10.** Coordinates and survey dates of the 21 parks.

| Park ID | Park | Code | Region | Longitude (°E) | Latitude (°N) | No. of plots | Survey period |
|---|---|---|---|---|---|---|---|
| 1 | Sanjiangkou Ecological Park | SJK | Inland | 119.3818 | 26.017 | 6 | 26 June 2026 |
| 2 | Dongjiangbin Park | DJB | Inland | 119.4137 | 26.0123 | 8 | 27 June 2026 |
| 3 | Yushan Park | YS | Inland | 119.3014 | 26.0804 | 4 | 25 June 2026 |
| 4 | Huaqiao Park | HQ | Inland | 119.2863 | 26.0468 | 4 | 23 June 2026 |
| 5 | Xihe Smart Sports Park | XH | Inland | 119.2516 | 26.079 | 4 | 22 June 2026 |
| 6 | Jinniushan Park | JNS | Inland | 119.2563 | 26.081 | 4 | 24 June 2026 |
| 7 | Minjiang Park South Garden | MJN | Inland | 119.2597 | 26.066 | 4 | 21 June 2026 |
| 8 | Dongdaihu Park | DDH | Coastal | 119.4892 | 25.9382 | 4 | 3 July 2026 |
| 9 | Bingxin Park | BX | Coastal | 119.5048 | 25.9673 | 4 | 5 July 2026 |
| 10 | Nanshan Park | NS | Coastal | 119.5205 | 25.9514 | 7 | 6 July 2026 |
| 11 | Jiangjunshan Park | JJS | Coastal | 119.6772 | 26.0176 | 6 | 30 June 2026 |
| 12 | Binhai New Town Sports Park | BH | Coastal | 119.5958 | 25.8724 | 5 | 1 July 2026 |
| 13 | Zhenghe Park | ZH | Coastal | 119.5145 | 25.9636 | 4 | 4 July 2026 |
| 14 | Changle People's Park | RM | Coastal | 119.4921 | 25.9357 | 4 | 2 July 2026 |
| 15 | 70 Nautical Miles Railway Coast Park | HCHA | Island | 119.8042 | 25.5164 | 5 | 28 May 2026 |
| 16 | Wanbao Park | WB | Island | 119.7835 | 25.4972 | 4 | 30 May 2026 |
| 17 | Ruyihu Park | RYH | Island | 119.6969 | 25.4794 | 5 | 31 May 2026 |
| 18 | Cuiyuan Park | CY | Island | 119.7911 | 25.5054 | 4 | 1 June 2026 |
| 19 | Lianhuashan Park | LHS | Island | 119.7913 | 25.5205 | 6 | 26 May 2026 |
| 20 | Sculpture Garden | DSY | Island | 119.7418 | 25.5049 | 5 | 29 May 2026 |

https://doi.org/10.3390/biology15211946

| Park ID | Park | Code | Region | Longitude (°E) | Latitude (°N) | No. of plots | Survey period |
|---|---|---|---|---|---|---|---|
| 21 | Longwangtou Ocean Park | LWT | Island | 119.8033 | 25.5035 | 5 | 27 May 2026 |

Note: Coordinates are in WGS 84. Park codes correspond to the plot codes used in the main text (e.g., plots MJN2 and NS7). Plot counts sum to 34 per region and 102 in total. All parks were surveyed between 26 May and 6 July 2026, one park per day, following the regional order Island, Inland and then Coastal.
